# Cyclopropanone: Preparation, Rotational Spectroscopy, and Semi-Experimental Equilibrium (*r*_*e*_^SE^) Structure

**DOI:** 10.1021/jacs.5c23052

**Published:** 2026-03-30

**Authors:** W. Hazel Styers, Brian J. Esselman, Samuel A. Wood, P. Bryan Changala, Gregory H. Jones, Michael C. McCarthy, John F. Stanton, R. Claude Woods, Robert J. McMahon

**Affiliations:** # Department of Chemistry, University of Wisconsin–Madison, Madison, Wisconsin 53706, United States; ‡ Center for Astrophysics | Harvard & Smithsonian, 60 Garden Street, Cambridge, Massachusetts 02138-1516, United States; § 172322 JILA, University of Colorado Boulder and National Institute of Standards and Technology, Boulder, Colorado 80309, United States; ∥ Department of Physics, University of Colorado Boulder, Boulder, Colorado 80309, United States; ⊥ Quantum Theory Project, Departments of Physics and Chemistry, 3463 University of Florida, Gainesville, Florida 32611, United States

## Abstract

We measured and analyzed the microwave (11–35 GHz) and millimeter-wave (85–750 GHz) rotational spectra of the normal isotopologue of cyclopropanone and nine additional isotopologues. The ability to perform experimental measurements is predicated on the synthesis of stable oligomers (or polymers) of cyclopropanone that release the monomeric form under vacuum to enable the investigation of cyclopropanone by gas-phase rotational spectroscopy. The spectral data provided in this work establish the foundation for radioastronomical searches for cyclopropanone. In particular, the new low-frequency microwave observations provide hyperfine-resolved rotational transitions that are necessary for radioastronomical observations at the low temperatures that characterize many cold interstellar molecular clouds. The extensive isotopic data set allows the first complete semiexperimental equilibrium (*r*_*e*_^SE^) structure of cyclopropanone. The 10 isotopologues used in this structure determination provide 30 moments of inertia. As a result, the values of all six independent structural parameters are highly converged, establishing a new experimental benchmark for the structure of this archetypal organic molecule. The highly precise and accurate *r*_*e*_^SE^ structure was compared to a computed equilibrium (*r*_*e*_) structure at the CCSD­(T)/cc-pCV6Z level with additional corrections addressing finite basis set, higher-level electron correlation, and relativistic effects, as well as the diagonal Born–Oppenheimer correction. The computed *r*_*e*_ structure systematically deviates from the *r*_*e*_^SE^ structure, despite the high-level methodology utilized. This finding is interpreted as revealing a subtle, but real, challenge for theoretical chemistry in predicting molecular structure at the level of accuracy that is now attainable experimentally.

## Graphical abstract

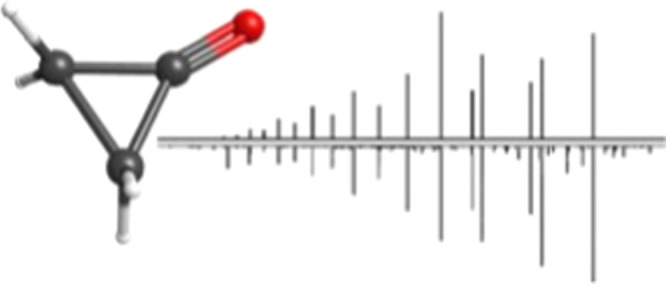

## Introduction

Cyclopropanone (Figure 1, *C*_2*v*_ C_3_H_4_O), the smallest cyclic aliphatic ketone, is a paradigm in organic chemistry because of its strained, three-membered ring.^1–3^ Cyclopropanone was proposed as a reactive intermediate well before its first detection by infrared spectroscopy.^4^ The unusually high frequency of the C=O stretch (1905 cm^–1^), relative to those of larger cyclic aliphatic ketones, provided direct evidence of ring strain.^4^ Early theoretical work^5,6^ suggested that the cyclopropanone ring was too strained for the molecule to exist as a cyclic species and favored a ring-opened (oxyallyl diradical) form (Figure 2).^6,7^ The structure of cyclopropanone as a cyclic species, however, was firmly established by analysis of its microwave spectrum by Pochan, Baldwin, and Flygare.^7–9^ The partial substitution structure (*r*_*s*_) clearly revealed the impact of ring strain on the structure of cyclopropanone: severely constricted angles (∠C2–C1–C3 = 64° at the nominal *sp* ^2^ center and ∠C1–C2–C3 = 57° at the nominal *sp* ^3^ center), a short C1–C2 bond (1.475 Å), and a long C2–C3 bond (1.575 Å).^8^ The origin of the long C2–C3 bond was later rationalized on the basis of the ultraviolet photoelectron spectrum.^10^ Computational studies of the C_3_H_4_O potential energy surface^11–13^ place the singlet oxyallyl diradical (ca. 30 kcal/mol) and allene oxide (ca. 12 kcal/mol) higher in energy than cyclopropanone (Figure 2). Negative-ion photoelectron spectroscopy established that the ring closure of singlet oxyallyl to cyclopropanone is barrierless.^14^ Several theoretical studies probed (and debated) the strain energy in cyclopropanone through comparisons with larger cyclic ketones,^15^ oxiranone,^16^ or other C_3_H_4_O isomers.^10,17^ Ring strain in cyclopropanone leads to its observed high propensity to react with nucleophiles and to polymerize.^1–3^

**Figure 1:**
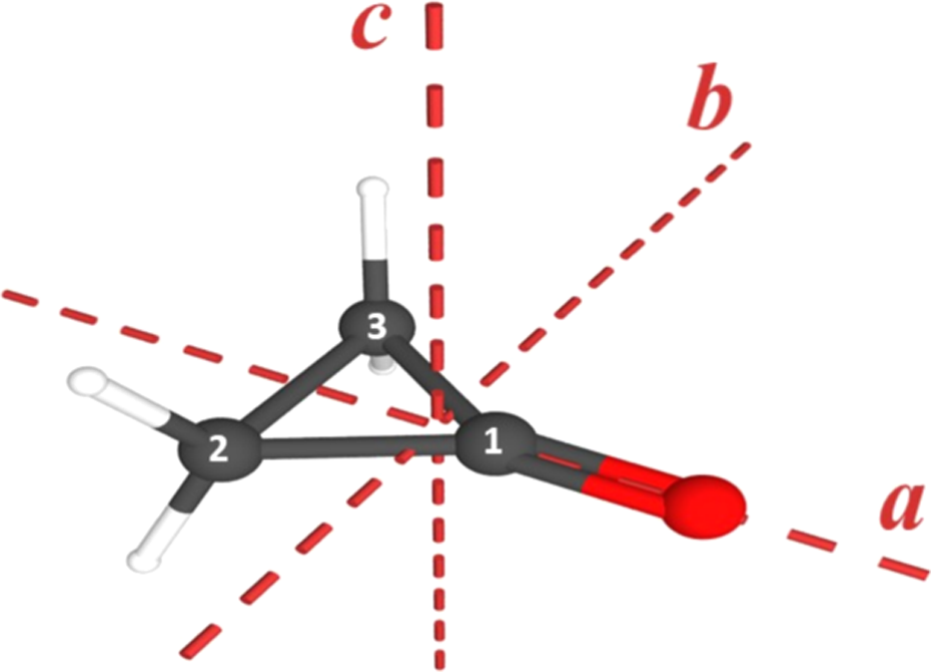
Cyclopropanone (C_3_H_4_O, *C*_2*v*_, μ_*a*_ = 2.67 (10) D, κ = –0.7767)^7^ with principal inertial axes and atom numbering.

**Figure 2:**
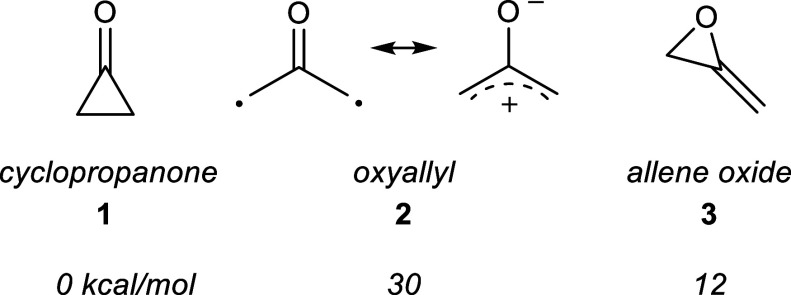
Structures and computed relative energies of several C_3_H_4_O isomers (CASPT2N).^11^

Our interest in cyclopropanone also stems from the relevance of this species to contemporary studies in astrochemistry. Two other C_3_H_4_O isomers have been detected in interstellar space.^18^ Propenal (H_2_C=CH–CHO; acrolein) has been detected in Sagittarius B2­(N),^19^ the galactic center,^20^ and IRAS 16293-2422 B.^21^ Methylketene (CH_3_–CH = C=O) was recently detected in TMC-1.^22^ Both isomers are lower in energy than cyclopropanone by ca. 21 kcal/mol.^12^ In laboratory experiments simulating the interaction of ionizing radiation with interstellar ices in cold molecular clouds, Kaiser and co-workers observed the formation of both propenal and cyclopropanone upon irradiation of ethylene/carbon monoxide ices with energetic electrons.^23^

Experimental investigations of cyclopropanone have been greatly hampered by the lack of convenient preparative methods and by its high reactivity. As a consequence, few spectroscopic studies of cyclopropanone have been reported since the seminal study by Pochan, Baldwin, and Flygare.^7–9^ The current investigation builds upon a fortuitous discovery that poly­(cyclopropanone) releases its monomeric form under vacuum at room temperature–a development that enables long-overdue experimental studies. Herein, we utilize rotational spectroscopy in conjunction with high-level quantum chemical calculations to determine the first accurate and precise semiexperimental equilibrium (*r*_*e*_^SE^) structure for this archetypal molecule. The *r*_*e*_^SE^ structure provides important benchmarks for computational chemistry. This spectroscopic study dramatically increases the range of measured rotational transitions available for cyclopropanone, which will support astronomical searches for this species.

## Methods

### Synthesis

As is common with small, reactive organic species, the synthesis and isolation of the unsubstituted parent molecule of a class is challenging. A typical preparation of cyclopropanone involves the reaction of ketene and diazomethane (Scheme 1).^1–3,24,25^ Ketene is used in excess, ensuring the concentration of diazomethane is low and minimizing reaction of diazomethane with the newly forming cyclopropanone to produce cyclobutanone.^26^ Although the yield of cyclopropanone is high in low-temperature solution, purification and isolation of neat (monomeric) cyclopropanone in appreciable quantity is highly problematic and typically results in polymerization.^24,25,27^ The polymer (Scheme 1), which is analogous to the paraformaldehyde polymer of formaldehyde, has not been extensively studied. The unexpected and remarkable finding in the current investigation is that this polymer (or oligomer) can serve as a reservoir of monomeric cyclopropanone. This characteristic enables the detailed spectroscopic studies described in this article.

**Scheme 1:**
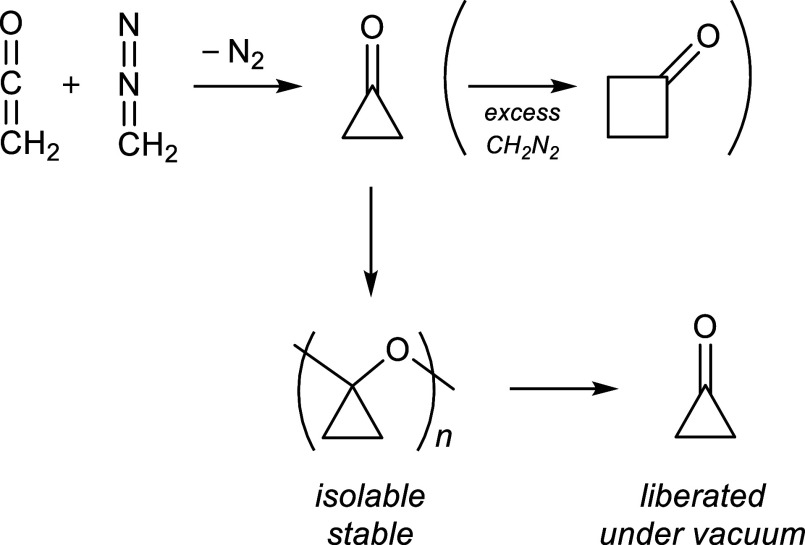
Preparation of Cyclopropanone

[2,2,3,3-^2^H]-Cyclopropanone was prepared from ketene-*d*_2_ and diazomethane-*d*_2_. The mixed isotopologues [2-^2^H]-, [2,2-^2^H]-, *trans*-[2,3-^2^H]-, *cis*-[2,3-^2^H]-, and [2,2,3-^2^H]-cyclopropanone were prepared from ketene-*d*_*x*_ and diazomethane-*d*_*x*_, with both reagents having approximately 50% deuterium incorporation. In each case, solid material was isolated from the reaction mixture, as described in Supporting Information. We attempted to characterize the material using standard analytical methods in polymer chemistry, but our efforts were thwarted by its insolubility. As a result, the degree of oligomerization/polymerization resulting from our preparation is unknown. Literature reports describe the polymer as being soluble in benzene and chloroform,^24^ but the material we obtained was not. One rationalization for this observation is that the material obtained from our synthesis has a higher degree of polymerization, hence lower solubility. In any case, subjecting the solid sample to high vacuum at room temperature releases monomeric cyclopropanone into the gas phase at a convenient rate. This fact is unequivocally established by the measurement of the rotational spectra reported in this work. The solid samples are stable and reproducibly release monomer, even months after preparation. This characteristic enables the sample to be stored and shipped. Full synthetic details, including safety considerations, are provided in Supporting Information.

### Spectroscopy

Rotational spectra were recorded from synthesized samples of cyclopropanone and cyclopropanone-*d*_*x*_. Millimeter-wave spectra (85–750 GHz) were recorded using a broadband spectrometer at the University of Wisconsin–Madison that has been described previously.^28^ Cyclopropanone was introduced into the gas chamber at room temperature as it was released from the polymer under vacuum. The backing pressure of cyclopropanone generated from the polymer (or by the monomer), and controlled through a needle valve, was sufficient to provide constant pressures of 15 to 30 mTorr in the instrument. The spectrum was collected at room temperature automatically over approximately 14 days (∼55 GHz per day), given the following experimental parameters: 0.6 MHz/s sweep rate, 10 ms time constant, and 50 kHz AM and 500 kHz FM modulation in a tone-burst design.^29^ Experimental frequency uncertainty of 50 kHz was assumed for all new millimeter-wave measurements. For previously published transition frequencies, the reported uncertainties were used.^7–9^ Millimeter-wave spectral files were combined, visualized, and analyzed using the AABS software package.^30,31^ Spectral prediction and least-squares fitting were carried out using Pickett’s SPFIT/SPCAT^32^ with Kisiel’s PIFORM, PISLIN, PLANM, and AC programs for spectroscopic analysis.^33^

Microwave spectra (5–40 GHz) were measured at the Center for Astrophysics in a cavity Fourier-transform microwave (FTMW) spectrometer equipped with a coaxial supersonic expansion source.^34^ Approximately 35 mg of solid poly­(cyclopropanone) was placed in a heated sample holder directly upstream of the pulsed solenoid valve, which seeded molecules into neon carrier gas at a total backing pressure of 2.5 kTorr. The sample was heated with 0.6 W of heater power (approximately 30 to 40 °C) to increase the vapor pressure of cyclopropanone monomer. The strong 2_0,2_ ← 1_0,1_ rotational transition of the normal isotopologue was measured with a signal-to-noise ratio (S/N) of ∼100 in 1 min integration time at a 5 Hz repetition rate. This S/N was sufficiently high to measure the ^18^O and both singly substituted ^13^C isotopologues at natural abundance. The *d*_4_ isotopologue was measured using a perdeuterio poly­(cyclopropanone) precursor. Five to seven pure rotational transitions (*J*″ ≤ 3, *K*_a_″ ≤ 1) were measured for each isotopologue. The S/N and instrumental resolution were sufficient to nearly fully resolve the hyperfine structure due to ^1^H-nuclear spin–spin and spin-rotation coupling, as well as ^2^H-nuclear quadrupole coupling. Experimental frequency uncertainties between 2 and 5 kHz were assumed for all new microwave measurements depending on the degree of unresolved quadrupole coupling. The hyperfine parameters were used to estimate the hyperfine-free rest frequency of the higher-frequency microwave transitions, which exhibit partially to fully collapsed hyperfine structure. The parameters in a theoretical model were least-squares fit/optimized to reproduce the hyperfine splittings using PGOPHER.^35^ Fourier-transform microwave spectral files were visualized and analyzed using PGOPHER.^35^

### Computation

The computational methods used in this work are similar to those used in several recent *r*_*e*_^SE^ structure determinations.^28,36–45^ A development version of CFOUR^46^ was used to optimize the structure at the CCSD­(T) level of theory. Calculations using cc-pVDZ, cc-pVTZ, or cc-pVQZ basis sets^47,48^ were performed with the frozen-core (fc) approximation. Calculations using cc-pCVTZ, cc-pCVQZ, cc-pCV5Z, and cc-pCV6Z basis sets^49^ included correlation of all electrons (ae). A “best theoretical estimate” (BTE) for the structure of cyclopropanone uses the CCSD­(T)/cc-pCV6Z structure as a basis, with four corrections that are evaluated using conventional methodology:^50–52^

1. Residual basis set effects beyond cc-pCV6Z.
2. Residual electron correlation effects beyond the CCSD­(T) treatment.
3. Effects of scalar (mass-velocity and Darwin) relativistic effects.
4. The diagonal Born–Oppenheimer correction to the fixed nucleus approximation.

The equations used to calculate these corrections, the computational methodology, the values of these corrections, and all output files are available in the Supporting Information. The methodology is similar to that used by us in earlier studies,^28,36–45^ but the treatment of electron correlation and relativistic effects has been improved through the use of better basis sets.

A second-order vibrational perturbation (VPT2) anharmonic frequency calculation, evaluating the cubic force constants using analytical second derivatives at displaced points based on the CCSD­(T)/cc-pCVTZ optimized structure, provided vibration–rotation interaction constants (*B*_0_–*B*_*e*_) and quartic and sextic centrifugal distortion constants for each isotopologue. Rotational *g*-tensor calculations provided electron-mass corrections for each isotopologue. The centrifugal distortion constants were applied directly in the least-squares fits of spectroscopic data for isotopologues whose constants could not be determined. The vibration–rotation interaction and electron-mass correction constants were used in the *xrefit* module of CFOUR to correct the experimental determinable rotational constants in the least-squares fitting of the semiexperimental equilibrium structure. The *xrefiteration* routine^39^ was used as a metric to identify isotopologues with problematic data sets and to assess the impact of the individual isotopologues on the convergence of the *r*_*e*_^SE^ data set to the final *r*_*e*_^SE^ structural parameters and statistical uncertainties.

Nuclear spin-rotation constants (*C*_*ii*_/kHz) were calculated at the CCSD­(T)/cc-pVTZ level of theory; nuclear spin–spin constants (*D*_*ii*_/kHz) were calculated using the *r*_*e*_^SE^ structure; nuclear quadrupole coupling constants for cyclopropanone-*d*_4_ (χ_*aa*_ = –0.0817 MHz, χ_*bb*_–χ_*cc*_ = –0.1527 MHz) were calculated at the CCSD­(T)/cc-pVQZ level of theory.^53^

## Results and Discussion

### Rotational Spectroscopy

In a technically challenging experiment,^7–9^ Pochan, Baldwin, and Flygare prepared cyclopropanone in the gas phase and measured 15 microwave transitions between 8 and 41 GHz – struggling to identify the target species amid a substantial background spectrum of acetone (their ketene precursor).^9^ That study also provided the first determination of its dipole moment via Stark effect, μ_*a*_ = 2.67 (10) D.^7^ Four transitions of the [1-^13^C] isotopologue and six transitions of the [2-^13^C] isotopologue were measured in natural abundance. The only deuterium-containing isotopologue to be studied was [2,2-^2^H]-cyclopropanone, for which seven transitions were measured. The ground-state rotational constants of four isotopologues were used to obtain a partial *r*_*s*_ structure, which determined the position of the oxygen atom using the center of mass and first-moment equations.^8^ To the best of our knowledge, no further studies of the rotational spectroscopy of cyclopropanone have been published since 1969.

In our measurements, we find that the rotational spectrum of the normal isotopologue of cyclopropanone is dominated by ^*a*^R_0,1_ transitions. No new Q-branch transitions were observed in this work. Q-branch transitions from the previous microwave work^8^ were included in our least-squares fit. The R-branch bands of transitions with constant *J*″ values of cyclopropanone exhibit the expected prolate-top behavior (Figure 3) with a 5:3 intensity alternation between even- and odd-*K*_*a*_ transitions, due to ^1^H-nuclear spin statistics. The *K*_*a*_ = 0^+^/1^–^ transitions are not degenerate below 260 GHz but form a degenerate pair of transitions (sharing the same *K*_*c*_ value) at higher frequency. The remaining low-*K*_*a*_ transitions are all nondegenerate with very large spacings–on the order of several GHz for very low-*K*_*a*_ (≤5) transitions–between *K*_*a*_^+^ and *K*_*a*_^–^ transitions with the same *K*_*c*_. As *K*_*a*_ increases, the transitions initially progress to higher frequency, lose intensity, and eventually turn around at moderate *K*_*a*_ values and then progress to lower frequency as *K*_*a*_ increases further. At high values of *K*_*a*_, the *K*_*a*_^+^ and *K*_*a*_^–^ transitions with the same *J*″ value become degenerate with different values of *K*_*c*_. The result is the easily identifiable step pattern of moderate-to-high-*K*_*a*_ transitions for a given *J*″ that increase in intensity as frequency increases (shown Figure 3 for *J*″ = 26), with interspersed low-*K*_*a*_ transitions from other R-branch bands. For the band shown, the transitions are nondegenerate between *K*_*a*_ = 0 and 10, with some of the nondegenerate transitions outside of the displayed range. The high-*K*_*a*_ band origins of the R branches are separated throughout the spectrum by approximately *B*_0_ + *C*_0_ (∼13.3 GHz) and *K*_*a*_ = 0 transitions are separated by 2*C*_0_ (∼11.6 GHz).

**Figure 3:**
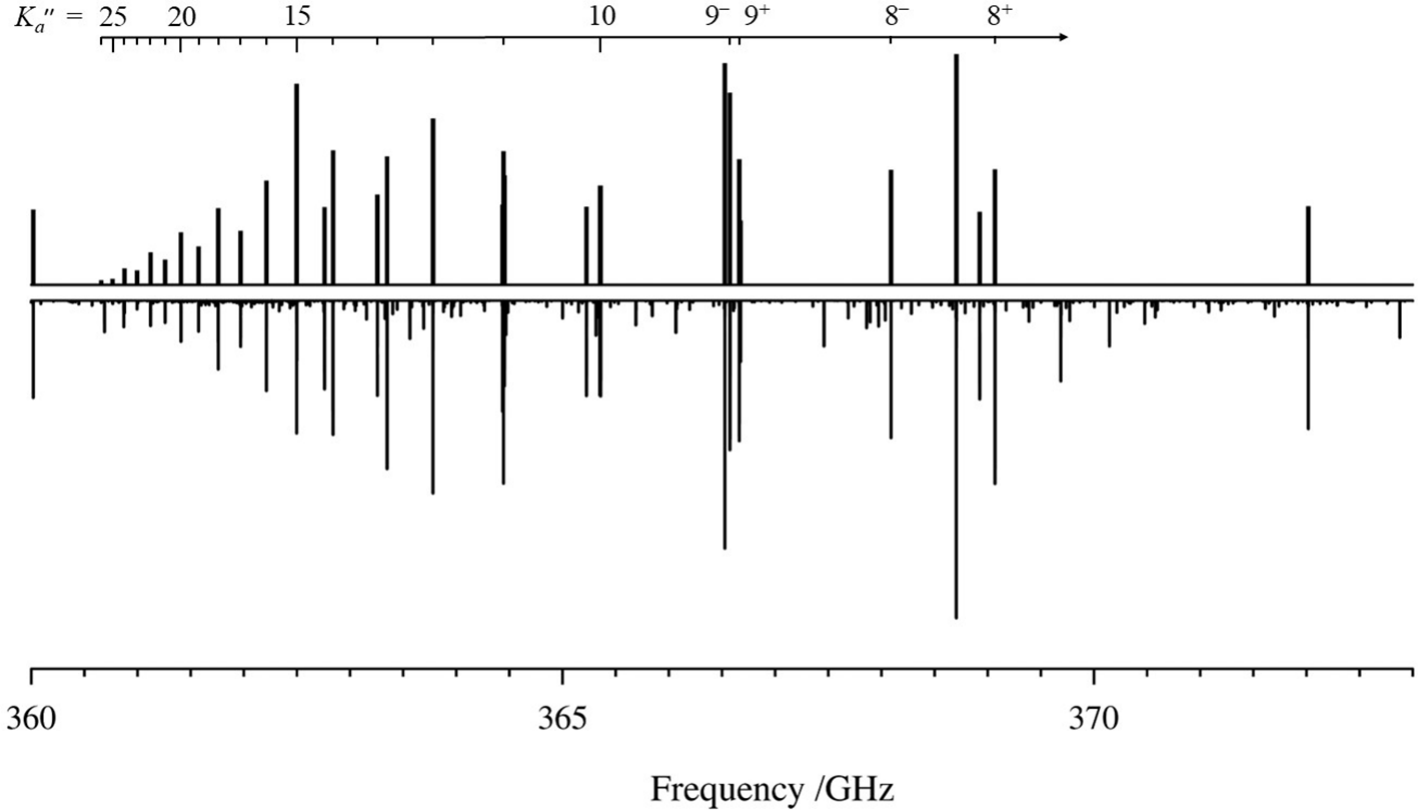
Experimental (bottom) and predicted (top) rotational spectra of the normal isotopologue of cyclopropanone from 360 to 373 GHz. Transitions assigned to heavy-atom isotopologues are not visible on this scale. Black tick marks designate the corresponding *K*_*a*_″ transitions of the *J*″+1 = 27 band. Many assigned and unassigned transitions from vibrationally excited states are present in the experimental spectrum and will be analyzed in future work.

The relatively small size of the cyclopropanone molecule and resultant large rotational constants give rise to a rotational spectrum that is quite sparse compared to larger molecules. Nearly 1400 transitions were measured for the vibrational ground state of the normal isotopologue with *J*″ ranging from 0 to 48 and *K*_*a*_″ from 0 to 33 (Figure 4). These transitions have been least-squares fit to sextic centrifugally distorted-rotor Hamiltonian models, using both the A and S reductions in the I^*r*^ representation. The resulting A-reduction spectroscopic constants are provided in Table 1, alongside those determined previously and those predicted computationally. The previous rotational constants from Pochan et al.^7–9^ show generally good agreement with the more precise values determined in this work. The largest relative discrepancy between the previously and newly determined values is for *A*_0_, whose previous value is nonetheless within 0.01% of that determined here.

**Figure 4:**
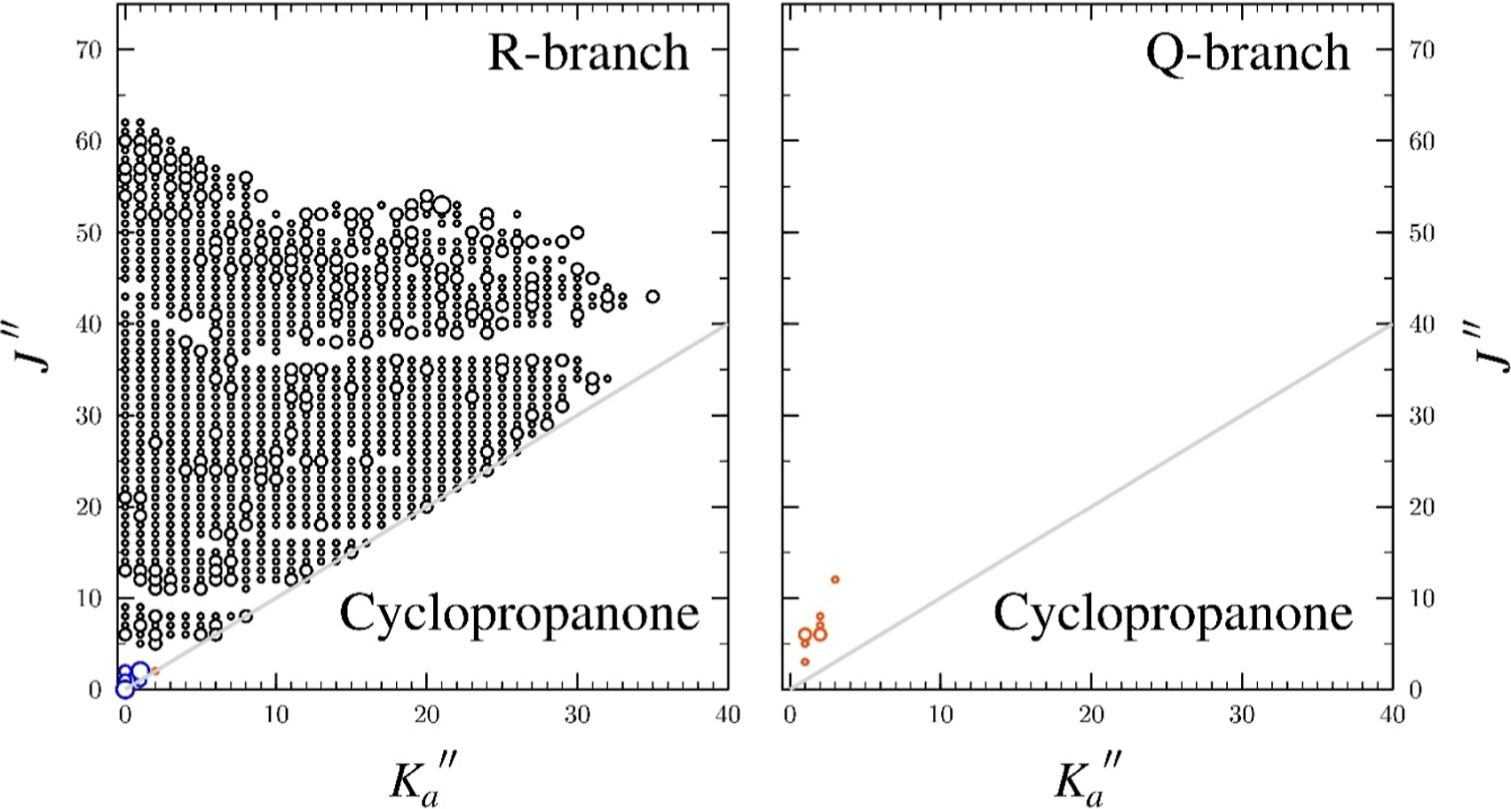
Data distribution plot for the least-squares fit of spectroscopic data for the vibrational ground state of cyclopropanone, normal isotopologue. The size of the symbol is proportional to the value of |(*f*_obs_–*f*_calc._)/δ*f*|, where δ*f* is the frequency measurement uncertainty of the relevant transition, and all quotient values are smaller than 3. Black circles represent millimeter-wave data, blue symbols represent microwave transitions recorded by the FT-microwave spectrometer, and orange symbols represent microwave data from Pochan et al.^8^

**Table 1:** Spectroscopic Constants of Cyclopropanone Isotopologues (A Reduction, I^*r*^ Representation)^a^

	normal isotopologue			[1-^13^C]	[2-^13^C]	[^18^O]
	Pochan et al.^b^	CCSD(T)^c^	Current Work			
*A*_0_ (MHz)	20155.11 (39)	20073	20153.365 8 (30)	20152.655 (76)	19682.209 5 (84)	20153.302 (79)
*B*_0_ (MHz)	7466.52 (4)	7419	7466.628 73 (14)	7457.536 42 (71)	7370.731 17 (47)	7017.393 17 (32)
*C*_0_ (MHz)	5871.85 (4)	5836	5871.865 29 (12)	5866.269 76 (65)	5772.367 49 (39)	5590.309 77 (32)
Δ _*J*_ (kHz)		1.791	1.881 587 (70)	1.821 64 (28)	1.835 32 (19)	1.571 (35)
Δ _*JK*_ (kHz)		16.95	17.180 41 (39)	17.612 (25)	16.536 3 (11)	[15.724 7]
Δ _*K*_ (kHz)		20.79	21.992 (22)	[20.948]	[20.079 9]	[22.222 9]
δ_*J*_ (kHz)		0.413	0.443 817 (39)	[0.413 759]	0.438 786 (99)	[0.352 88]
δ_*K*_ (kHz)		8.767	9.191 5 (20)	[8.728 26]	8.988 7 (49)	[8.200 37]
Φ _*J*_ (Hz)		0.000 694 5	0.000 786 (17)	[0.000 697]	[0.000 666 7]	[0.000 565 7]
Φ _*JK*_ (Hz)		0.028 82	0.029 15 (74)	[0.028 105 5]	[0.027 125 3]	[0.026 326 8]
Φ _*KJ*_ (Hz)		0.104 8	0.114 5 (26)	[0.104 285 8]	[0.100 068 8]	[0.096 508]
Φ _*K*_ (Hz)		–0.180 7	[−0.180 7]	[−0.179 5]	[−0.172 3]	[−0.169 8]
ϕ_*J*_ (Hz)		0.000 342 3	0.000 394 3 (88)	[0.000 344 2]	[0.000 329 9]	[0.000 278 5]
ϕ_*JK*_ (Hz)		0.009 317	0.010 47 (47)	[0.009 114 3]	[0.009 104]	[0.008 349 6]
ϕ_*K*_ (Hz)		0.288 6	0.336 8 (90)	[0.287 522 8]	[0.278 198 4]	[0.280 419]
κ^d^	–0.776 7	–0.778	–0.776 7	–0.777 2	–0.770 2	–0.804 0
*P*_*cc* 0_ (uÅ^2^)^e^	3.346 20 (42)	3.350	3.346 903 (2)	3.347 559 (48)	3.345 586 (7)	3.346 053 (49)
*N*_lines_ ^f^	15		1390^g^	30 ^*g*^	263 ^*g*^	5
σ (MHz)			0.035	0.069	0.053	0.000 22

	[2-^2^H]	[2,2-^2^H]	*trans*-[2,3-^2^H]	*cis*-[2,3-^2^H]	[2,2,3-^2^H]	[2,2,3,3-^2^H]
*A*_0_ (MHz)	18412.848 2 (37)	17000.008 2 (64)	16808.405 6 (41)	16886.993 7 (41)	15575.747 3 (32)	14415.943 4 (47)
*B*_0_ (MHz)	7216.211 81 (23)	6960.856 95 (31)	7009.942 62 (33)	6988.301 08 (34)	6773.413 65 (29)	6574.506 83 (29)
*C*_0_ (MHz)	5659.297 39 (21)	5478.084 46 (27)	5455.105 42 (28)	5467.078 71 (27)	5291.658 57 (43)	5132.229 75 (26)
Δ _*J*_ (kHz)	1.788 633 (73)	1.658 472 (89)	1.668 140 (90)	1.688 737 (91)	1.547 64 (26)	1.423 99 (13)
Δ _*JK*_ (kHz)	15.802 80 (57)	14.198 67 (72)	15.487 97 (46)	14.457 35 (44)	13.722 09 (34)	12.758 40 (67)
Δ _*K*_ (kHz)	[15.228 1]	11.363 (45)	[8.427 3]	[11.649 9]	[6.287 9]	3.293 (24)
δ_*J*_ (kHz)	0.419 264 (43)	0.390 198 (49)	0.387 805 (56)	0.390 365 (56)	0.359 100 (49)	0.330 397 (44)
δ_*K*_ (kHz)	7.629 2 (18)	5.461 9 (19)	6.964 8 (18)	6.307 9 (18)	5.026 2 (14)	3.937 7 (12)
Φ _*J*_ (Hz)	[0.000 774 9]	[0.000 608 7]	[0.000 6451]	[0.000 953 8]	0.000 549 (71)	0.000 411 (38)
Φ _*JK*_ (Hz)	[0.031 570 4]	0.024 91 (29)	[0.039 68]	[0.025 34]	[0.026 71]	0.022 00 (25)
Φ _*KJ*_ (Hz)	0.076 45 (82)	0.057 11 (97)	[0.019 13]	[0.059 44]	[0.035 77]	0.042 78 (52)
Φ _*K*_ (Hz)	[−0.109 2]	[−0.073 14]	[−0.069 48]	[−0.091 26]	[−0.064 57]	[−0.058 56]
ϕ_*J*_ (Hz)	[0.000 353 2]	[0.000 280 9]	[0.000 287 6]	[0.000 401 6]	[0.000 279 9]	[0.000 24]
ϕ_*JK*_ (Hz)	[0.010 07]	[0.005 561]	[0.013 07]	[0.007 254]	[0.006 207]	[0.003 394]
ϕ_*K*_ (Hz)	[0.212 2]	[0.097 10]	[0.172 1]	[0.158 5]	[0.089 41]	[0.050 14]
κ^d^	–0.755 8	–0.742 6	–0.726 1	–0.733 6	–0.711 8	–0.689 3
*P*_*cc* 0_ (uÅ^2^)^e^	4.090 130 (4)	5.038 228 (7)	4.759 166 (5)	4.902 284 (5)	5.776 925 (6)	6.727 409 (7)
*N*_lines_ ^f^	634	658^g^	491	491	624	626
σ (MHz)	0.035	0.060	0.045	0.045	0.042	0.045

a Values in square brackets held fixed at the CCSD­(T)/cc-pCVTZ predicted value in the least-squares fit. Values in parentheses represent 2σ experimental uncertainties.

b *B*_0_ values and uncertainties reported by Pochan et al.^8^.

c *B*_0_ values and distortion constants computed using CCSD­(T)/cc-pCVTZ.

d Calculated using PLANM from the *B*_0_ constants.

e *P*_*cc*_ = – (*I*_*c*_–*I*_*a*_–*I*_*b*_)/2 = – 1/2 Δ _*i* 0_, calculated using PLANM.

f Number of independent transitions.

g Includes transitions reported by Pochan et al.^8^.

The relative sparsity of the ground-state spectrum, along with a lack of intense transitions from low-energy vibrationally excited states (lowest-energy fundamental ∼317 cm^–1^),^54^ allowed for easy observation and assignment of [1-^13^C]- and [2-^13^C]-isotopologue transitions at natural abundance across the observed frequency regions. The [^18^O]-isotopologue was observed at natural abundance only in the microwave frequency range. Deuterium-substituted species were not observed at natural abundance, but all six possible cyclopropanone-*d*_*x*_ isotopologues were observed in deuterium-enriched samples. None of the individual deuterium-containing isotopologues were present at sufficiently high partial pressure to allow its [^13^C]- or [^18^O]-isotopologues to be detected. Transitions for isotopologues were assigned using previously reported spectroscopic constants, where available,^7,8^ or from computed constants. Loomis-Wood plots were used to assign the low-*K*_*a*_ series, allowing for refinement of the spectroscopic constants and assignment of additional transitions. Transition data sets for the isotopologues were least-squares fit to both A- and S-reduction models. The A-reduction spectroscopic constants are provided in Table 1, with S-reduction spectroscopic constants, computed spectroscopic constants, data distribution plots, and a detailed discussion of the constants provided in the Supporting Information.

The normal isotopologue of cyclopropanone displayed partially resolved ^1^H-nuclear spin-rotation and spin–spin coupling splitting in lowest rotational transition 1_0,1_ ← 0_0,0_ (Figure 5). The hyperfine splitting was observed, albeit only in this transition, due to the improved resolution compared to previous work.^7–9^ The hyperfine-split line profile was simulated using computed nuclear spin-rotation constants combined with nuclear spin–spin coupling constants derived from the ultimate *r*_*e*_^SE^ structure (Table 2). This simulation is in good agreement with the partially resolved measurement (Figure 5). The hyperfine splitting patterns are predominantly determined by the spin–spin coupling interactions between the closest pairs of ^1^H atoms because this coupling parameter scales as *r* ^–3^, where *r* is the internuclear distance. Owing to the large number of independent hyperfine parameters (nine total), a direct experimental optimization was poorly determined, and we report only the theoretical values and those derived from the *r*_*e*_^SE^ structure (Table 2). The averaged, hyperfine-free line center was used in the least-squares fitting. Deuterium-nuclear quadrupole coupling was observed in the low-frequency spectrum (Figure 6) for several low-*J* and low-*K*_*a*_ transitions. From these transitions, only χ_*aa*_ was well determined (χ_*aa*_ = –0.078 (1) MHz) while χ_*bb*_–χ_*cc*_ was held constant at its computed value (−0.1527 MHz).

**Figure 5:**
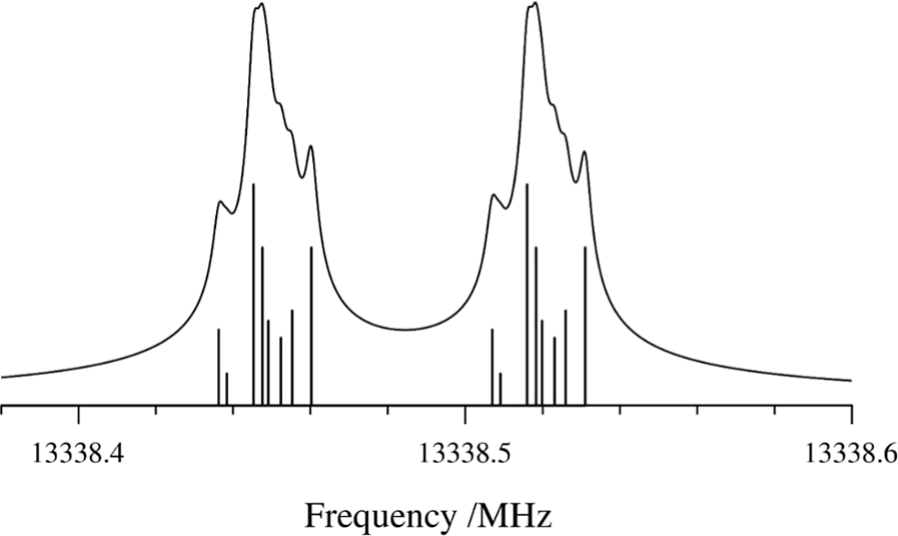
Experimental 1_0,1_ ← 0_0,0_ transition of the normal isotopologue of cyclopropanone. Simulated hyperfine-splitting components arising from ^1^H-nuclear spin-rotation and spin–spin hyperfine structure are superimposed along the frequency axis. The larger Doppler doublet splitting arises from the coaxial cavity-expansion geometry. The experimental spectrum is the result of 16 min of integration.

**Table 2:** Computed Hyperfine Parameters of Cyclopropanone^a^

	CCSD(T)
*C*_*aa*_	–1.91
*C*_*bb*_	–0.60
*C*_*cc*_	–0.18
*D*_*aa*_ (HCH)	19.68
*D*_*bb*_ (HCH)	19.68
*D*_*cc*_ (HCH)	–39.36
*D*_*aa*_ (HCCH, *cis*)	7.18
*D*_*bb*_ (HCCH, *cis*)	–14.36
*D*_*cc*_ (HCCH, *cis*)	7.18
*D*_*aa*_ (HCCH, *trans*)	3.87
*D*_*bb*_ (HCCH, *trans*)	–3.81
*D*_*cc*_ (HCCH, *trans*)	–0.05

a Nuclear spin-rotation constants (*C*_*ii*_/kHz) calculated at the CCSD­(T)/cc-pVTZ level of theory; nuclear spin–spin constants (*D*_*ii*_/kHz) calculated using the *r*_*e*_^SE^ structure. “HCH” refers to the geminal hydrogen atoms on a common carbon atom. “HCCH, *cis*” refers to vicinal hydrogen atoms on the same side of the heavy-atom plane. “HCCH, *trans*” refers to vicinal hydrogen atoms on opposite sides of the heavy-atom plane.

**Figure 6:**
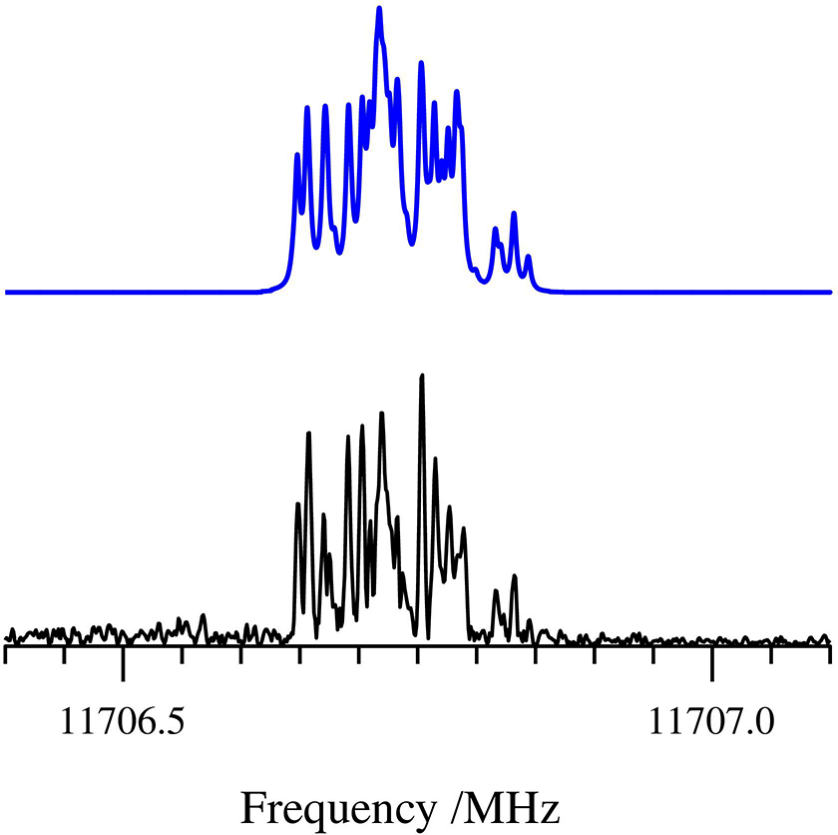
1_0,1_ ← 0_0,0_ transition of [2,2,3,3-^2^H]-cyclopropanone, showcasing deuterium quadrupole coupling due to four identical quadrupolar nuclei. Experimental spectrum (bottom, black); simulated fit using χ_*aa*_ = –0.078 MHz, χ_*bb*_–χ_*cc*_ = –0.1527 MHz (top, blue). The experimental spectrum is the result of 242 min of integration.

### Semi-Experimental Equilibrium Structure

The *r*_*e*_^SE^ structure of cyclopropanone was determined in an analogous manner to that used in our previous works.^28,36–45^ The ground-state rotational constants determined for both the A and the S reductions (*B*_0_^(A)^ and *B*_0_^(S)^) were converted into determinable constants (*B*_0_″^(A)^ and *B*_0_″^(S)^), which removes the effects of centrifugal distortion.^55^ The determinable constants and the equations used to calculate them are provided in Supporting Information. The averaged values of the determinable rotational constants (*B*_0_″^(A)^ and *B*_0_″^(S)^) of each isotopologue were employed in *xrefit*, along with the vibration–rotation interaction and electron-mass distribution corrections computed at the CCSD­(T)/cc-pCVTZ level of theory. The *xrefit* module of CFOUR converts these values to the equilibrium moments of inertia and determines the equilibrium structural parameters by least-squares fitting.

The semiexperimental equilibrium structural parameters for cyclopropanone, determined by least-squares fitting the 30 moments of inertia from 10 isotopologues with the vibration–rotation interaction and electron-mass corrections, are given in Figure 7 and Table 3. Table 3 also provides the previous partial-substitution structure (*r*_*s*_),^7–9^ the CCSD­(T) BTE *r*_*e*_ structure, and the CCSD­(T)/cc-pCV6Z *r*_*e*_ structure. An inherent limitation of high-resolution structure determination is the assumption that the equilibrium molecular structure does not change upon isotopic substitution (Born–Oppenheimer approximation). We recently presented evidence that the precision of current semiexperimental equilibrium structure determination is sufficiently high to challenge this basic assumption. Through careful analysis of benzene-*h*_6_ vs benzene-*d*_6_ and ketene-*h*_2_ vs ketene-*d*_2_, we estimated that the C–H bond is slightly longer than the C–D bond at a nominally *sp* ^2^-hybridized carbon atom (0.000 07 Å for benzene,^43^ 0.000 06 Å for ketene^28,43^ ). That the C–H/C–D bond distances differ in the fifth decimal place but become convoluted in the *r*_*e*_^SE^ structure determination involving many deuterium-containing isotopologues suggested to us that the accuracy of any *r*_*e*_^SE^ bond distance measurement (involving H) is limited to the fourth decimal place (i.e., 0.0001 Å). The precision to which the bond angles can be determined is similarly impacted by the breakdown of Born–Oppenheimer approximation, though in a manner that is less straightforward to quantify. These considerations are the reason that we provide a recommended *r*_*e*_^SE^ structure with all bond lengths rounded to the fourth decimal place and bond angles/dihedral angles to the third decimal place (Table 3).

**Figure 7:**
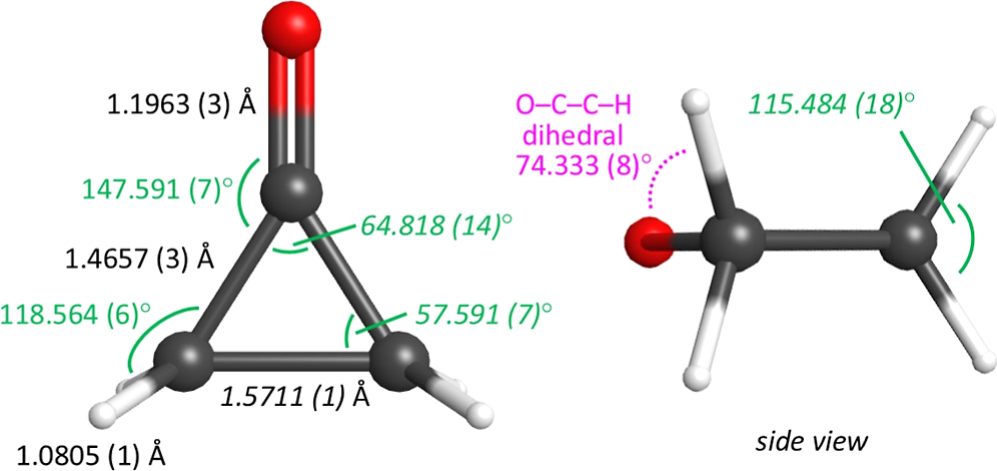
Semiexperimental equilibrium structure (*r*_*e*_^SE^) of cyclopropanone determined from 10 isotopologues. 2σ statistical uncertainties in parentheses. Bond distances (black), angles (green), and dihedral angle (pink) shown. Italicized value indicates that the parameter was determined in an alternate Z-matrix or by mathematical relationships with propagated uncertainty.

**Table 3:** Semi-Experimental Equilibrium (*r*_*e*_^SE^) and Computed (*r*_*e*_) Structural Parameters for Cyclopropanone^a^ ^,^ ^b^ ^,^ ^c^

	*r*_*s*_	*r*_*e*_^SE^	*r*_*e*_^SE^	CCSD(T) BTE^e^		CCSD(T)/cc-pCV6Z	
	Pochan et al.^8^ ^,^ ^d^	current work	**recommended**	**value**	*obs.–calc.*	value	*obs.–calc.*
*R*_C1–O_	1.191 (20)	1.196 30 (35)	**1.1963 (3)**	**1.196 26**	0.000 04	1.195 65	0.000 65
*R*_C1–C2_	1.475 (17)	1.465 66 (27)	**1.4657 (3)**	**1.465 91**	–0.000 25	1.465 67	–0.000 01
*R*_C2–H_	1.086 (25)	1.080 539 (41)	**1.0805 (1)**	**1.080 81**	–0.000 27	1.080 66	–0.000 12
*R*_C2–C3_	1.575 (12)	1.571070 (44)	**1.5711 (1)**	**1.570 73**	0.000 34	1.570 01	0.001 06
θ_C2–C1–O_	147.7 (4)	147.5910 (68)	**147.591 (7)**	**147.609**	–0.018	147.616	–0.025
θ_C1–C2–H_		118.5637 (59)	**118.564 (6)**	**118.550**	0.013	118.548	0.015
θ_H–C2–C1–O_		74.3331 (85)	**74.333 (8)**	**74.288**	0.045	74.290	0.043
θ_C1–C2–C3_	57.7 (4)	57.5910 (68)	**57.591 (7)**	**57.609**	–0.018	57.616	–0.025
θ_C2–C1–C3_	64.6 (8)	64.818 (14)	**64.818 (14)**	**64.781**	0.036	64.768	0.050
θ_C3–C2–H_		117.1612 (42)	**117.161 (4)**	**117.183**	0.022	117.182	0.021
θ_H–C2–H_	114 (2)	115.484 (18)	**115.484 (18)**	**115.468**	0.017	115.462	0.012
*N*_isotopologues_	4	10	**10**				

a Distances in Angstrom (Å), angles in degree (°). Values in parentheses represent 2σ experimental uncertainties.

b Values in boldface facilitate comparison of best available experimental and computational data.

c Values in italics are dependent parameters determined in an alternate *Z*-matrix or by mathematical relationships with propagated statistical uncertainty.

d Uncertainties reported in ref 8 are 1σ.

e CCSD­(T)/cc-pCV6Z optimized structure with BTE corrections applied (see text).

### Equilibrium Structure–Comparison of Experiment and Theory

Figure 8 displays the *r*_*e*_^SE^ value of each structural parameter with its 2σ uncertainty, enabling comparison with computed *r*_*e*_ parameters (CCSD­(T) methodology using several different basis sets) and with the value that we refer to as the Best Theoretical Estimate (BTE). In numerous recent *r*_*e*_^SE^ structure determinations, we find that, regardless of the size of the electronic structure calculation, the agreement between the *r*_*e*_^SE^ structural parameters and those predicted by theory is always improved by including the corrections as noted above to provide the BTE.^28,36–45^ In the majority of cases studied, the values of the BTE parameters fall within the uncertainty of the *r*_*e*_^SE^ values. The striking finding, in the case of cyclopropanone, is that most of the structural parameters for cyclopropanone do not display the expected measure of agreement between *r*_*e*_^SE^ values (derived from experiment and theory) and *r*_*e*_ BTE (purely theoretical) values. This situation raises the question as to whether the discrepancy lies with experiment, or theory, or both (*vide infra*).

**Figure 8:**
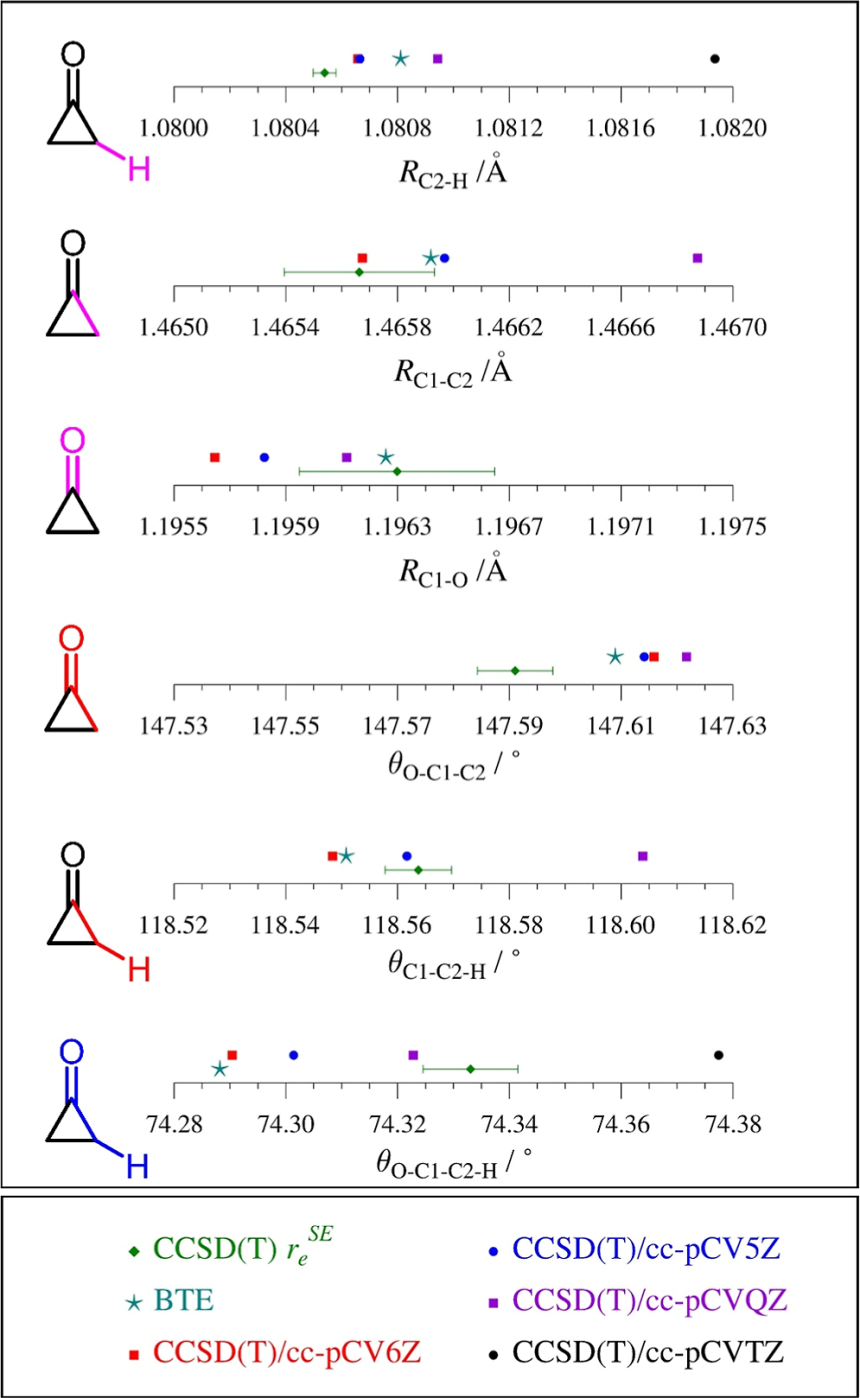
Graphical comparison of the cyclopropanone structural parameters with bond distances in Ångstrom (Å) and angles in degrees (°). The CCSD­(T)/cc-pCVTZ parameter values (black) do not fall within the scale provided, except for the values of θ_O–C1–C2–H_. and *R*_C2–H_. The statistical uncertainties for *r*_*e*_^SE^ parameters are 2σ.

There are some anomalies in the computational data presented in Figure 8. Most of the computed structural parameters show the appropriate behavior with respect to increasing basis-set size from cc-pCVTZ to cc-pCV6Z, *i*.*e*., successive values of the parameter converge toward an asymptote as the basis-set size increases. The parameters θ_C1–C2–H_ and θ_O–C1–C2_, however, do not display such convergence. For θ_C1–C2–H_, the change from TZ to QZ is smaller than the change from QZ to 5Z (TZ data point does not fall within the range shown in Figure 8). Because only QZ, 5Z, and 6Z geometries were used in the basis-set extrapolation that is applied in the BTE analysis, this anomaly is not likely to be a significant complication. For θ_O–C1–C2_, while the parameters decrease from TZ to QZ to 5Z, the 6Z value is slightly larger than the 5Z value. The change between the 6Z and 5Z values (−0.0017°) is much smaller than the discrepancy between the BTE and experimental parameter for θ_O–C1–C2_ (0.017°), indicating a similarly nonsignificant anomaly. A two-point extrapolation was employed for θ_O–C1–C2_ using the 5Z and 6Z values. None of the BTE values for the two bond angles and the dihedral angle falls within the 2σ statistical uncertainty of the corresponding *r*_*e*_^SE^ parameter. In each of those cases, the BTE correction moves the parameter closer to the experimental value, but not far enough to fall within 2σ. Regarding bond distances, the BTE values of *R*_C1–O_ and *R*_C1–C2_ parameters fall within the 2σ statistical uncertainty of the corresponding *r*_*e*_^SE^ parameter. For *R*_C2–H_, the lack of agreement between the *r*_*e*_^SE^ and BTE values is perhaps a consequence of the exceedingly small uncertainty of the *r*_*e*_^SE^ parameter (Figure 8). While it is clear that not all parameters have converged at the 6Z level, improvement to the basis set extrapolation alone will likely not improve the agreement between theory and experiment. Alternately, the disagreement between the *r*_*e*_^SE^ and BTE structures may be caused by inadequate theoretical corrections applied to experimental *B*_0_ constants. The *B*_0_ constants themselves are expected to be as accurate as they are precise. The applied corrections could introduce systematic errors in the structural parameters that would not be reflected in their 2σ statistical uncertainties. Although there are other possibilities, a strong candidate for the source of such a systematic error is the omission of higher-order vibration–rotation interaction terms.

### Isotopologue Dependence of Structure Determination

A minimum requirement for gas-phase structure determination by rotational spectroscopy is single isotopic substitution at each unique atom. In our recent studies, we find that using as many isotopologues as possible facilitates the determination of highly accurate and precise structures.^28,36–45^ The current semiexperimental structure for cyclopropanone, which includes 10 isotopologues that provide 30 moments of inertia for the determination of six independent structural parameters, is substantially overdetermined. The minimum set of isotopologues (core set) needed for a full *r*_*e*_^SE^ structure determination of cyclopropanone includes five species: the normal isotopologue and its [1-^13^C]-, [2-^13^C]-, [^18^O]-, and [2-^2^H]-isotopologues. Even this core set provides enough information (15 independent moments) to “over-determine” the structure. To probe the impact of including additional isotopologues in the structure determination, we utilize the *xrefiteration* routine.^39^ The routine begins by determining an *r*_*e*_^SE^ structure on the basis of the core set (single isotopic substitution at each position) and then sequentially recomputes an *r*_*e*_^SE^ structure by adding the isotopologue that produces the largest decrease in the overall statistical uncertainty of the structural parameters. In all previous applications of this script,^28,37–45^ the analysis revealed a decrease in the uncertainty of the *r*_*e*_^SE^ structural parameters upon inclusion of isotopologues beyond the core set. The *xrefiteration* analysis for cyclopropanone is qualitatively different. Even using only the core set, the values of the uncertainties are very low. Moreover, inclusion of additional isotopologues beyond the core set does not decrease the relative statistical uncertainties of the structural parameters (Figure 9). This behavior indicates that the *r*_*e*_^SE^ structure is not improving in an obvious way with the additional structural information provided by the deuterium-containing isotopologues. In fact, inclusion of additional isotopologues in the data set causes a subtle increase in statistical uncertainty, particularly those in the bond distances. This effect has also been observed in some data sets with a very large number of isotopologues, and we previously speculated that this subtle increase in uncertainty may arise because of the difference in bond length between C–H and C–D bonds (see also discussion that follows).^44,45^

**Figure 9:**
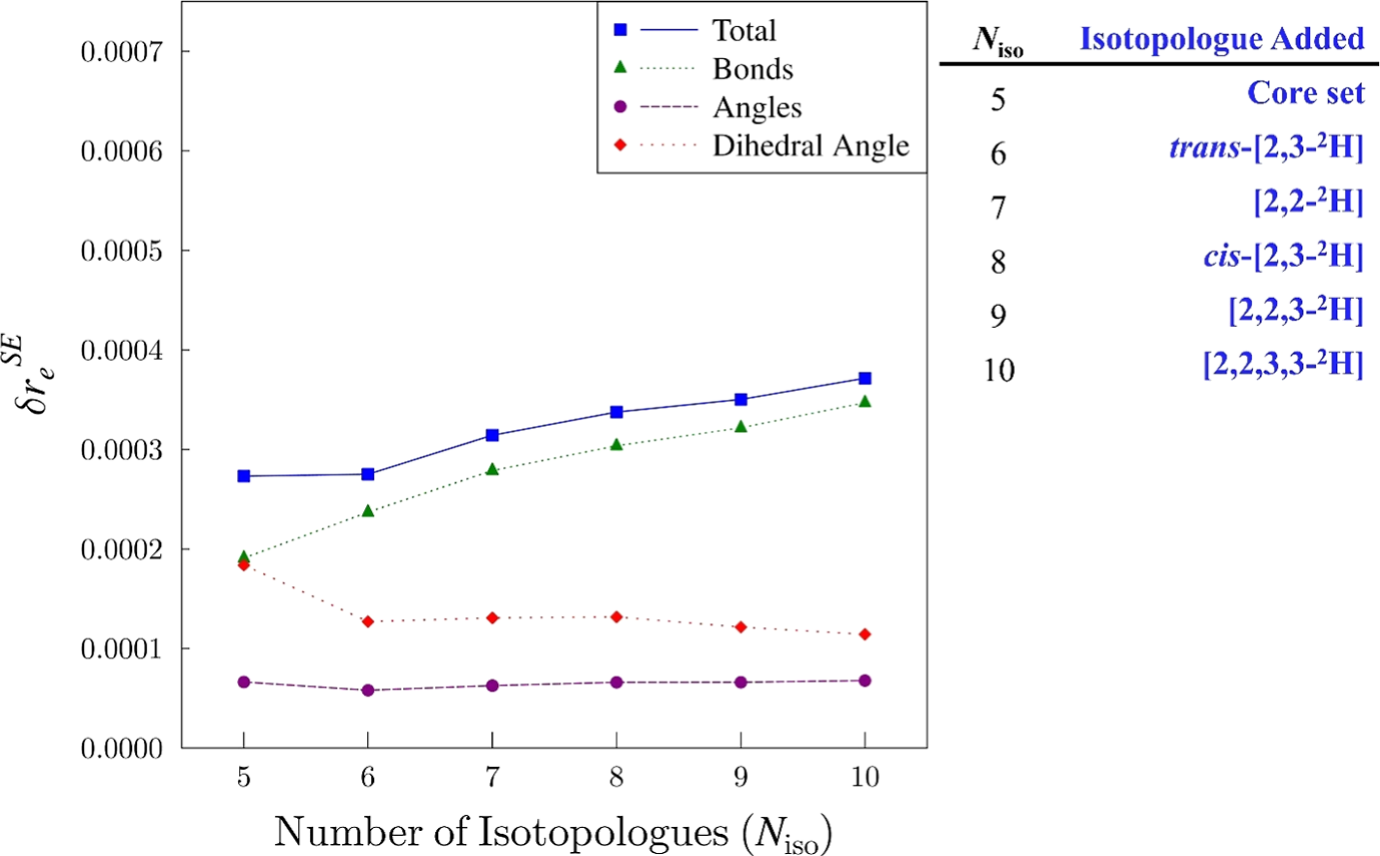
Plot of *r*_*e*_^SE^ *Uncertainty* (δ*r*_e_^SE^) as a function of the number of isotopologues (*N*_iso_) incorporated into the structure determination data set for cyclopropanone. The total relative statistical uncertainty (δ*r*_e_^SE^ total, blue squares), the relative statistical uncertainty in the bond distances (δ*r*_e_^SE^ bonds, green triangles), the relative statistical uncertainty in the dihedral angle (δ*r*_e_^SE^ dihedral angle, red diamonds), and the relative statistical uncertainty in the angles (δ*r*_e_^SE^ angle, purple circles) are presented. Statistical uncertainties reported as 2σ. The *y*-axis scale is chosen so that it is comparable to *xrefiteration* plots published previously.

The impact of each isotopologue beyond the core set on the *r*_*e*_^SE^ structural parameters of cyclopropanone is depicted in Figure 10. Statistical uncertainties decrease for some parameters and increase slightly for others as more isotopologues are added to the *r*_*e*_^SE^ structure. The values of the parameters, however, are nearly constant from the core set of five isotopologues to the ten used in the *r*_*e*_^SE^ structure presented in this work. The cyclopropanone structure appears to be converged even when using only the minimal data set of single-substitution isotopologues in the structural least-squares fit (Figure 10). That the cyclopropanone structure is converged strongly suggests that incorporation of additional isotopologues is unlikely to result in any further improvement to the *r*_*e*_^SE^ structure. It could not have been known *a priori* that additional isotopologues beyond the core set would have so little impact on the cyclopropanone *r*_*e*_^SE^ geometry.

**Figure 10:**
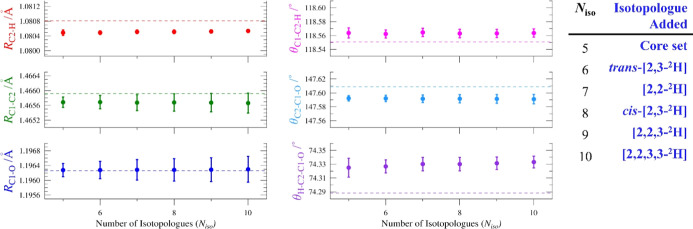
Plots of the *r*_*e*_^SE^ structural parameters as a function of the number of isotopologues (*N*_iso_) and their 2σ uncertainties, with consistent scales for each distance (0.0015 Å) and each angle (0.08°). The dashed line in each plot is the BTE value calculated for that parameter. The isotopologue ordering along the *x*-axis is the same as that in Figure 9.

The structures of ketene^28^ and hydrazoic acid^40^ display convergence in their determined structural parameters, with small statistical uncertainties similar to those of cyclopropanone. In those cases, however, the *r*_*e*_^SE^ and *r*_*e*_ BTE parameters agree within the 2σ uncertainties of the experimental values,^28,40^ indicating that the small values of 2σ are not solely responsible for the different behavior. The accuracy of the experimentally determined rotational constants also does not appear to be the origin of the discrepancy. The quality of the fit of a rotational spectrum, or a series of spectra, may be assessed by consideration of the inertial defect ( Δ _*i*_) for a planar species or second moment (*P*_*ii*_) for a nonplanar species. Given the very close agreement of the second moments (*P*_*cc*_) for all-protio isotopologues (normal, [1-^13^C]-, [2-^13^C]-, [^18^O]-) (Table 4) and the very close agreement of the *P*_*bb*_ values for the species that differ only by isotopic substitution on the *C*_2*v*_ axis (normal, [1-^13^C], [^18^O]) (Table 4), it is unlikely that the rotational constants used to determine the *r*_*e*_^SE^ structure could be substantially improved. This analysis is discussed in greater detail in Supporting Information.

**Table 4:** Second Moments (*P*_*bb e*_) of Cyclopropanone Isotopologues with Vibration-Rotation and Electron-Mass Corrections^a^

isotopologue	*P*_*aa e*_ (uÅ^2^)	*P*_*bb e*_ (uÅ^2^)	*P*_*cc e*_ (uÅ^2^)	*c*_H_ (Å)^b^
C_3_H_4_O	64.1403	21.4023	3.365 31	0.913 671
[1-^13^C]	64.2281	21.4024	3.365 34	0.913 675
[2-^13^C]	65.0205	21.9980	3.365 31	0.913 671
[^18^O]	68.4671	21.4023	3.365 16	0.913 650
[2-^2^H]	65.7319	23.0271	4.103 64	
[2,2-^2^H]	67.3345	24.3581	5.046 50	
*trans*-[2,3-^2^H]	67.1130	24.9709	4.768 85	
*cis*-[2,3-^2^H]	67.1905	24.6931	4.910 94	
[2,2,3-^2^H]	68.5950	26.3309	5.781 37	
[2,2,3,3-^2^H]	69.8874	27.9840	6.727 59	0.913 817

a *P*_*bb*_ = – (*I*_*b*_–*I*_*a*_–*I*_*c*_)/2, where *P*_*aa*_ and *P*_*cc*_ can be determined by the appropriate permutation of this equation. Second moments calculated using *I*_*e*_ values, which were obtained by applying corrections for vibration–rotation interaction and electron mass distribution to *I*_0_ values.

b *c*-coordinate of the H/D atom; *c*_H_ = (*P*_*cc*_/4*m*_H_)^1/2^ = distance from the *a,b*-plane (heavy-atom plane); *m*_H_ = 1.007825035 amu for ^1^H, 2.014101779 amu for ^2^H.

### C–H Bond Distance

The apparent C–H bond distance in cyclopropanone (1.0805 Å) determined in the current semiexperimental (*r*_*e*_^SE^) structure is similar to the highly precise values recently reported for benzene (1.0809 Å) and ketene (1.0759 Å). Although the nominal hybridization at C2 in cyclopropanone may be considered to be *sp* ^3^, angle strain in the three-membered ring causes the C–C bonds to be enriched in *p*-orbital character (Walsh orbitals) and the C–H bonds to be rich in *s*-orbital character.^56,57^ Various metrics reveal the hybridization of C–H bonds in cyclopropanone to be *sp* ^2^;^56–58^ our studies now reinforce this interpretation by direct measurement of bond distance. The semiexperimental equilibrium C–H bond distance in cyclopropanone is slightly longer than that of cyclopropane (1.0805 Å vs 1.0786 Å), while the semiexperimental equilibrium H–C–H bond angle is slightly larger (115.484° vs 114.97°).^59,60^ These effects are likely correlated, arising from hyperconjugation of the σ_C–H_ orbitals with the π*_C–O_ orbital in cyclopropanone.

Also of interest to us are analyses that enable an experimental estimate of the apparent difference in C–H vs C–D bond distance. The methodology for semiexperimental structure determination cannot address this question, as the method is founded upon the assumption that molecular structure is independent of isotope (Born–Oppenheimer approximation). Indeed, the accuracy and precision of our measurements create a situation in which the difference in C–H vs C–D bond distance is one factor that defines the uncertainty of the measurements (see below). Structural analyses derived from the rotational constants/moments of inertia, however, provide some insight into the effects of isotopic substitution. In some small molecules with suitably high symmetry, such as ketene-*h*_2_/ketene-*d*_2_ and cyclopropanone-*h*_4_/cyclopropanone-*d*_4_, it is possible to determine certain distances (often nonbonding) involving H and D through consideration of second moments (*P*_*ii*_).^61^ In ketene, the two equivalent hydrogen atoms are the only masses with nonzero coordinates along the *b*-inertial axis. The distance of the mass from the *ac*-plane, *b*_H_, is given by *b*_H_ = (*P*_*bb*_/2*m*_H_)^1/2^ = [−(*I*_*b*_–*I*_*a*_–*I*_*c*_)/4*m*_H_ ]^1/2^.^55,62^ In cyclopropanone, the four equivalent hydrogen atoms are the only masses with nonzero coordinates along the *c*-inertial axis. The distance of the mass from the *ab*-plane, *c*_H_, is given by *c*_H_ = (*P*_*cc*_/4*m*_H_)^1/2^ = [−(*I*_*c*_–*I*_*a*_–*I*_*b*_)/8*m*_H_ ]^1/2^. As shown in Table 4, these coordinate values are determined at an order of magnitude higher precision than even the already precise *r*_*e*_^SE^ bond distances. These coordinates can be treated as proxies for the C–H bond distance and may be converted to the C–H bond distance by trigonometry using the respective *r*_*e*_^SE^ structural parameters, assuming they remain constant. For ketene, *b*_H_ = 0.940 45 Å, *b*_D_ = 0.940 32 Å; *R*_C–H_ = 1.076 17 Å, *R*_C–D_ = 1.076 02 Å (as estimated from second moments).^28^ In ketene, the difference in estimated bond lengths (*R*_C–H_–*R*_C–D_ = +0.000 15 Å) provided an independent assessment that the uncertainty in a semiexperimental value for a C–H bond distance should be conservatively reported as ± 0.000 2 Å.^28,43^ For cyclopropanone, *c*_H_ = 0.913 67 Å, *c*_D_ = 0.913 82 Å; *R*_C–H_ = 1.080 48 Å, *R*_C–D_ = 1.080 66 Å (as estimated from second moments; Table 4).^63^ This sign reversal, in which the C–H bond is shorter than the C–D bond (*R*_C–H_–*R*_C–D_ = –0.000 18 Å), is somewhat unexpected, although the value falls within the uncertainty (±0.000 2 Å) estimated in the ketene study. Various subtle factors may influence the result of this simplistic second-moment analysis and the interpretation of C–H vs C–D bond distance.^64–71^ In the framework of the Born–Oppenheimer approximation, the positions of the hydrogen atoms are, by definition, exactly the same in the true equilibrium structure, independent of isotopic substitution (*R*_C–H_ = *R*_C–D_ and *c*_H_ = *c*_D_). Thus, the differences must reflect either an actual breakdown in the Born–Oppenheimer approximation or a possible systematic inaccuracy in the *B*_*e*_ values employed, for reasons already discussed in the context of comparing *r*_*e*_^SE^ and BTE parameter values. Notably, the reported bond-distance isotope shift due to Born–Oppenheimer breakdown in the diatomic HCl vs DCl system, where it has been very well determined, is only −0.000 02 Å,^72^ (an order of magnitude less than the above differences for ketene and cyclopropanone). This suggests that either having multiple hydrogen atoms in a molecule has a cumulative effect or indeed other factors may be at play here, such as inadequate vibration–rotation interaction corrections. The full origin of the differences between the C–H and C–D bond-distance values that we obtain is yet to be determined.^73–79^

### Structural Comparisons

Molecular structures depicting the best available structural parameters for cyclopropanone, cyclopropane, and methylenecyclopropane are presented in Figure 8. An *sp* ^2^-hybridized center, which is even more demanding in terms of bond angle than an *sp* ^3^-hybridized center, causes a rather serious distortion in the equilateral ring of cyclopropane. The internal bond angle at the carbonyl carbon of cyclopropanone increases to 64.818° from the nominal value of 60° in cyclopropane. This deformation is accompanied by a decrease in the C1–C2 bond distance (1.4657 Å) and an increase in the C2–C3 bond distance (1.5711 Å), relative to cyclopropanone.^8,80^ These effects are more pronounced than in methylenecyclopropane (63.87°, C2–C3 bond distance 1.5415 Å; Figure 11).^80,81^ The strained C2–C1–C3 bond angle and the long C2–C3 bond of cyclopropanone are consistent with the known reactivity of cyclopropanone and its tendency to undergo nucleophilic addition at the carbonyl carbon, as well as many of ring–opening reactions across the C2–C3 bond, studied by Turro et al.^85–89^

**Figure 11:**
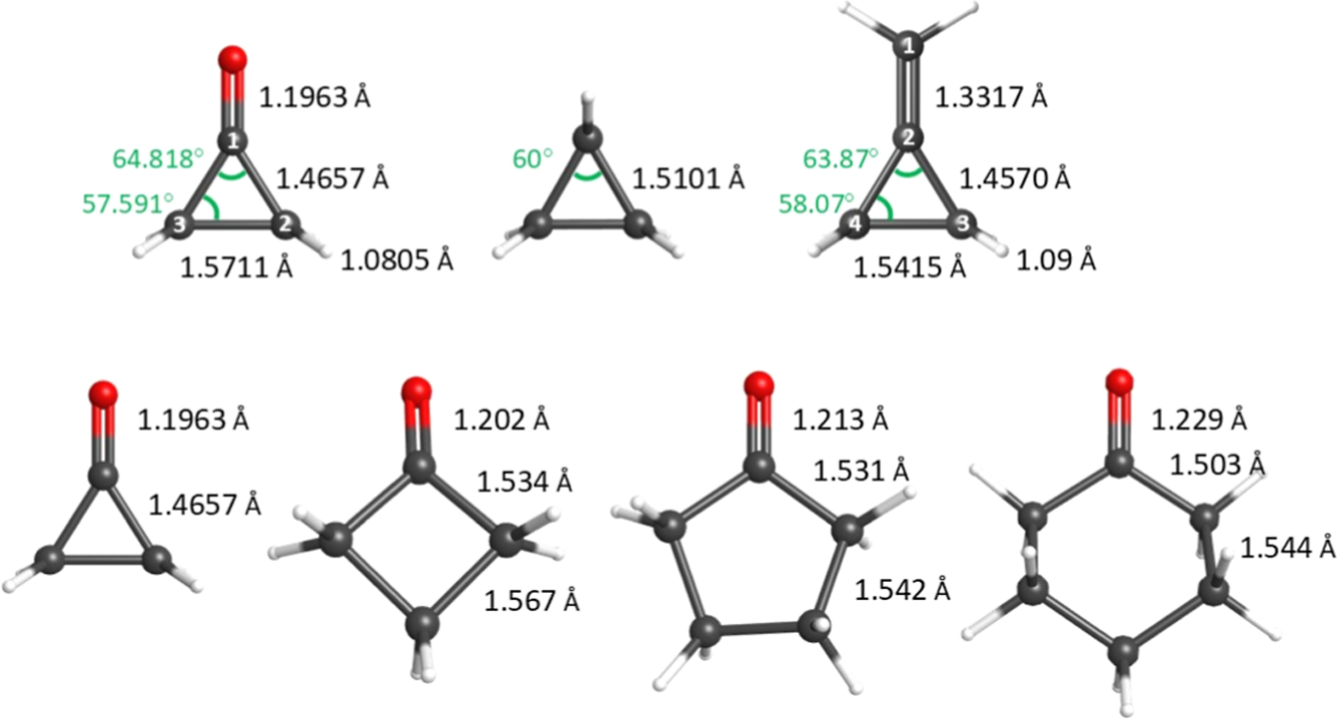
Cyclopropanone structural parameters (this work) compared to those of related molecules: cyclopropane,^59,60^ methylenecyclopropane,^81^ cyclobutanone,^82^ cyclopentanone,^83^ cyclohexanone.^84^

Figure 11 also provides the best available C–O bond distances for the four smallest cyclic aliphatic ketones: cyclopropanone, cyclobutanone,^82^ cyclopentanone,^83^ and cyclohexanone.^84^ The C–O bond length decreases with decreasing ring size. This trend is inversely correlated with the trend in the frequency of the gas-phase carbonyl stretching vibration: cyclopropanone (1906 cm^–1^),^4^ cyclobutanone (1814 cm^–1^),^82,90^ cyclopentanone (1775 cm^–1^),^91,92^ and cyclohexanone (1735 cm^–1^).^92,93^ (For reference, (CH_3_)_2_CO, an acyclic dialkylketone, displays a C–O bond distance of 1.214 Å^94^ and a gas-phase carbonyl stretching vibration of 1731 cm^–1^).^95^ The increase in vibrational frequency with decreasing ring size can be rationalized by an increase in the *p*-orbital character of the σ_C–C_ bonds in the ring, which increases the *s*-orbital character of the σ_C–O_ bond. This change in hybridization increases the C–O bond strength and increases the C–O bond vibrational frequency.^96^

## Conclusion

The development of new preparative methods enabled extensive gas-phase spectroscopic measurements of the highly reactive cyclopropanone molecule. The newly measured microwave and millimeter-wave transitions reported in this work provide the basis for future astronomical observations. Cyclopropanone is a likely candidate for detection in the interstellar medium, an assertion supported by the previous detection of cyclopropenone, the detection of other C_3_H_4_O isomers, and laboratory experiments that suggest the formation of cyclopropanone under astrophysical conditions. Laboratory measurements (10–750 GHz) now cover the majority of the frequency range available to modern radiotelescopes. The measurement of hyperfine-resolved transitions in the microwave region is important for astronomical detection in sources like molecular clouds, where the rotational temperature is very low, typically 10 K or colder. Computed spectroscopic constants are in close agreement with the experimental values determined in this work.

A highly precise and accurate semiexperimental equilibrium (*r*_*e*_^SE^) structure for cyclopropanone has been determined from the rotational spectra of 10 isotopologues, affording more than an order-of-magnitude improvement over the precision of the previously reported *r*_*s*_ structure. The best theoretical estimate (BTE) structure of cyclopropanone stands in contrast to those for other recently studied small molecules (hydrazoic acid^40^ and ketene^28^) because only two *r*_*e*_ structural parameters of cyclopropanone fall within 2σ of the corresponding semiexperimental values. This lack of agreement arises in part due to the level of precision of the cyclopropanone *r*_*e*_^SE^ parameters, as the magnitudes of the discrepancies in bond lengths and angles are still quite small (less than 0.0003 Å and 0.04°, respectively). A complete understanding of the discrepancy, however, cannot be attributed to the high precision of the *r*_*e*_^SE^ structure or the basis-set extrapolation explored in this work. Improvement to the basis-set extrapolation, alone, will likely not improve the agreement between theory and experiment, which strongly suggests that improvements to the theoretical corrections used to obtain the *r*_*e*_ structure and/or improvements to the corrections applied to obtain the BTE will be required to further narrow the gap. As such, this work provides both a benchmark and a challenge for future theoretical and computational work.

## Supplementary Material





## Notes

Equations used to calculate determinable constants, equations and corrections used to calculate the BTE, A-reduction and S-reduction spectroscopic constants, data distribution plots, discussion of spectroscopic constants and second moments, experimental details for synthesis and characterization of cyclopropanone oligomer/polymer and its isotopologues (PDF)
Least-squares fitting output files, computational output files, *xrefit* output files, *xrefiteration* output files (ZIP)

## †

Deceased March 21, 2025

## References

[1] Turro N. J. (1969). Cyclopropanones. Acc. Chem. Res..

[2] Wasserman, H. H. ; Berdahl, D. R. ; Lu, T.-J. The chemistry of cyclopropanones. In The Chemistry of the Cyclopropyl Group; Rappoport, Z. , Ed.; John Wiley & Sons, Ltd: Chichester, 1987; Vol. 1, pp 1455–1532. 10.1002/0470023449.ch23

[3] De Kimpe, N. Cyclopropanone. In Encyclopedia of Reagents for Organic Synthesis (EROS); John Wiley & Sons, Ltd., 2001. 10.1002/047084289X.rc302

[4] van
Tilborg W. J. M. (1973). The chemistry of small ring compounds. Part 22. Absorptions
of cyclopropanone in the infrared carbonyl region. Tetrahedron Lett..

[5] See footnote (8) of reference 7.

[6] Hoffmann R. (1968). Trimethylene
and the addition of methylene to ethylene. J.
Am. Chem. Soc..

[7] Pochan J. M., Baldwin J. E., Flygare W. H. (1968). Microwave
Spectrum, Structure, and
Dipole Moment in Cyclopropanone. J. Am. Chem.
Soc..

[8] Pochan J. M., Baldwin J. E., Flygare W. H. (1969). Microwave
Spectrum and Structure
of Cyclopropanone. J. Am. Chem. Soc..

[9] Pochan, J. M. Microwave Spectroscopic Studies; Cyclopropanone, Molecular Zeeman Effects, and Supersonic Nozzle Beam Applications; Ph.D. Dissertation; University of Illinois: Urbana, IL, 1969.

[10] Martino P.
C., Shevlin P. B., Worley S. D. (1977). The Electronic Structure of Cyclopropanone. J. Am. Chem. Soc..

[11] Hess B. A., Eckart U., Fabian J. (1998). Rearrangements of Allene
Oxide, Oxyallyl, and Cyclopropanone. J. Am.
Chem. Soc..

[12] Bermúdez C., Tercero B., Motiyenko R. A., Margulès L., Cernicharo J., Ellinger Y., Guillemin J. C. (2018). The millimeter-wave
spectrum of methyl ketene and the astronomical search for it. Astron. Astrophys..

[13] Rush L. A., Gallo K. F., Stumetz K. S., Rodríguez-Pérez I. A., Cremeens M. E. (2022). Non-statistical
dynamics for the allene oxide to cyclopropanone
conversion. J. Phys. Org. Chem..

[14] Ichino T., Villano S. M., Gianola A. J., Goebbert D. J., Velarde L., Sanov A., Blanksby S. J., Zhou X., Hrovat D. A., Borden W. T. (2009). The Lowest Singlet and Triplet States of the
Oxyallyl Diradical. Angew. Chem., Int. Ed..

[15] Bach R. D., Dmitrenko O. (2006). The Effect of Carbonyl Substitution
on the Strain Energy
of Small Ring Compounds and Their Six-Member Ring Reference Compounds. J. Am. Chem. Soc..

[16] Rodriquez C. F., Williams I. H. (1997). Ring Strain
Energy and Enthalpy of Formation of Oxiranone:
an *ab initio* Theoretical Determination. J. Chem. Soc., Perk. Trans..

[17] Lim D., Hrovat D. A., Borden W. T., Jorgensen W. L. (1994). Solvent
Effects on the Ring Opening of Cyclopropanones to Oxyallyls: A Combined
ab Initio and Monte Carlo Study. J. Am. Chem.
Soc..

[18] Müller H. S. P., Schlöder F., Stutzki J., Winnewisser G. (2005). The Cologne
Database for Molecular Spectroscopy, CDMS: a Useful Tool for Astronomers
and Spectroscopists. J. Mol. Struct..

[19] Hollis J. M., Jewell P. R., Lovas F. J., Remijan A., Møllendal H. (2004). Green Bank
Telescope Detection of New Interstellar Aldehydes: Propenal and Propanal. Astrophys. J..

[20] Requena-Torres M. A., Martín-Pintado J., Martín S., Morris M. R. (2008). The Galactic Center: The Largest
Oxygen-Bearing Organic
Molecule Repository. Astrophys. J..

[21] Manigand S., Coutens A., Loison J.-C., Wakelam V., Calcutt H., Müller H. S. P., Jørgensen J. K., Taquet V., Wampfler S. F., Bourke T. L. (2021). The ALMA-PILS survey: first detection of the
unsaturated 3-carbon molecules Propenal (C_2_H_3_CHO) and Propylene (C_3_H_6_) towards IRAS 16293–2422
B. Astron. Astrophys..

[22] Fuentetaja R., Bermúdez C., Cabezas C., Agúndez M., Tercero B., Marcelino N., Pardo J. R., Margulès L., Motiyenko R. A., Guillemin J. C. (2023). Discovery of CH_3_CHCO in TMC-1 with the QUIJOTE line survey. Astron. Astrophys..

[23] Abplanalp M. J., Borsuk A., Jones B. M., Kaiser R. I. (2015). On the Formation
and Isomer Specific Detection of Propenal (C_2_H_3_CHO) and Cyclopropanone (*c*-C_3_H_4_O) in Interstellar Model Ices *–* A Combined
FTIR and Reflection Time-of-Flight Mass Spectroscopic Study. Astrophys. J..

[24] Schaafsma S. E., Steinberg H., de Boer T. J. (1966). The Synthesis of Cyclopropanone. Recl. Trav. Chim. Pays-Bas.

[25] Turro N. J., Hammond W. B. (1966). Cyclopropanone. J. Am. Chem.
Soc..

[26] Lipp P., Köster R. (1931). Ein neuer
Weg zum Cyclobutanon. Ber. Dtsch. Chem. Ges.
A/B.

[27] Rodriguez H. J., Chang J.-C., Thomas T. F. (1976). Thermal,
Photochemical, and Photophysical
Processes in Cyclopropanone Vapor. J. Am. Chem.
Soc..

[28] Smith H. H., Esselman B. J., Wood S. A., Stanton J. F., Woods R. C., McMahon R. J. (2023). Improved Semi-Experimental
Equilibrium Structure and
High-Level Theoretical Structures of Ketene. J. Chem. Phys..

[29] Pickett H. M. (1980). Determination
of Collisional Linewidths and Shifts by a Convolution Method. Appl. Opt..

[30] Kisiel Z., Pszczółkowski L., Drouin B. J., Brauer C. S., Yu S., Pearson J. C., Medvedev I. R., Fortman S., Neese C. (2012). Broadband
Rotational Spectroscopy of Acrylonitrile: Vibrational Energies from
Perturbations. J. Mol. Spectrosc..

[31] Kisiel Z., Pszczółkowski L., Medvedev I. R., Winnewisser M., De Lucia F. C., Herbst E. (2005). Rotational
Spectrum of *trans–trans* Diethyl Ether in the
Ground and Three Excited Vibrational States. J. Mol. Spectrosc..

[32] Pickett H. M. (1991). The Fitting
and Prediction of Vibration-Rotation Spectra with Spin Interactions. J. Mol. Spectrosc..

[33] Kisiel, Z. PROSPE - Programs for Rotational Spectroscopy; http://info.ifpan.edu.pl/~kisiel/prospe.htm accessed 22-Mar-2026.

[34] Grabow J.-U., Palmer E. S., McCarthy M. C., Thaddeus P. (2005). Supersonic-jet cryogenic-resonator
coaxially oriented beam-resonator arrangement Fourier transform microwave
spectrometer. Rev. Sci. Instrum..

[35] Western C. M. (2017). PGOPHER:
A program for simulating rotational, vibrational and electronic spectra. J. Quant. Spectrosc. Radiat. Transfer.

[36] Heim Z. N., Amberger B. K., Esselman B. J., Stanton J. F., Woods R. C., McMahon R. J. (2020). Molecular Structure Determination:
Equilibrium Structure
of Pyrimidine (*m*-C_4_H_4_N_2_) from Rotational Spectroscopy (*r*
_e_
^SE^) and High-Level Ab Initio Calculation (*r*
_e_) Agree Within the Uncertainty of Experimental Measurement. J. Chem. Phys..

[37] Orr V. L., Ichikawa Y., Patel A. R., Kougias S. M., Kobayashi K., Stanton J. F., Esselman B. J., Woods R. C., McMahon R. J. (2021). Precise
Equilibrium Structure Determination of Thiophene (*c*-C_4_H_4_S) by Rotational Spectroscopy—Structure
of a Five-Membered Heterocycle Containing a Third-Row Atom. J. Chem. Phys..

[38] Esselman B. J., Zdanovskaia M. A., Owen A. N., Stanton J. F., Woods R. C., McMahon R. J. (2021). Precise
equilibrium structure of thiazole (*c*-C_3_H_3_NS) from twenty-four isotopologues. J. Chem. Phys..

[39] Owen A. N., Zdanovskaia M. A., Esselman B. J., Stanton J. F., Woods R. C., McMahon R. J. (2021). Semi-Experimental
Equilibrium (*r*
_e_
^SE^) and Theoretical
Structures of Pyridazine (*o*-C_4_H_4_N_2_). J. Phys. Chem. A.

[40] Owen A. N., Sahoo N. P., Esselman B. J., Stanton J. F., Woods R. C., McMahon R. J. (2022). Semi-Experimental Equilibrium (*r*
_e_
^SE^) and Theoretical Structures of
Hydrazoic Acid
(HN_3_). J. Chem. Phys..

[41] Zdanovskaia M. A., Esselman B. J., Kougias S. M., Amberger B. K., Stanton J. F., Woods R. C., McMahon R. J. (2022). Precise equilibrium structures of
1*H*- and 2*H*-1,2,3-triazoles (C_2_H_3_N_3_) by millimeter-wave spectroscopy. J. Chem. Phys..

[42] Bunn H. A., Esselman B. J., Franke P. R., Kougias S. M., McMahon R. J., Stanton J. F., Widicus
Weaver S. L., Woods R. C. (2022). Millimeter/Submillimeter-wave
Spectroscopy and the Semi-experimental Equilibrium (*r*
_e_
^SE^) Structure of 1*H*-1,2,4-Triazole
(c-C_2_H_3_N_3_). J. Phys. Chem. A.

[43] Esselman B. J., Zdanovskaia M. A., Owen A. N., Stanton J. F., Woods R. C., McMahon R. J. (2023). Precise
Equilibrium Structure of Benzene. J. Am. Chem.
Soc..

[44] Esselman B. J., Atwood M. G., Nguyen H. V. L., Stanton J. F., Woods R. C., McMahon R. J. (2025). Semi-Experimental
Equilibrium (*r*
_e_
^SE^) Structure
of Thiophene - Attempting to Resolve
the Heavy-Atom Problem with 46 Isotopologues. J. Chem. Phys..

[45] Zdanovskaia M. A., Esselman B. J., Kougias S. M., Atwood M. G., Jones G. H., Stanton J. F., Woods R. C., McMahon R. J. (2025). Precise Semi-Experimental
Equilibrium (*r*
_e_
^SE^) Structure
of Pyridine from 32 Isotopologues: Accurate Assessment of the Effect
of Nitrogen-Atom Substitution in Aromatic Rings. J. Phys. Chem. A.

[46] Stanton, J. F. ; Gauss, J. ; Cheng, M. E. ; Harding, M. E. ; Matthews, D. A. ; Szalay, P. G. ; with contributions from Asthana, A. ; Auer, A. A. ; Bartlett, R. J. ; Benedikt, U. ; Berger, C. ; Bernholdt, D. E. ; Blaschke, S. ; Bomble, Y. J. ; Christiansen, O. ; Datta, D. ; Engel, F. ; Faber, R. ; Greiner, J. ; Heckert, M. ; Heun, O. ; Hilgenberg, M. ; Huber, C. ; Jagau, T.-C. ; Jonsson, D. ; Jusélius, J. ; Kirsch, T. ; Kitsaras, M.-P. ; Klein, K. ; Kopper, G. M. ; Lauderdale, W. J. ; Lipparini, F. ; Liu, J. ; Metzroth, Monzel, L. ; Mück, L. A. ; Nottoli, T. ; O’Neill, D. P. ; Oswald, J. ; Price, D. R. ; Prochnow, E. ; Puzzarini, C. ; Ruud, K. ; Schiffmann, F. ; Schwalbach, W. ; Simmons, C. ; Stopkowicz, S. ; Tajti, A. ; Uhlířová, T. ; Vázquez, J. ; Wang, F. ; Watts, J. D. ; Yergün, P. ; Zhang, C. ; Zheng, X. and the integral packages *MOLECULE* (Almlöf, J.; Taylor, P. R.), *PROPS* (Taylor, P. R.), *ABACUS* (Helgaker, T.; Jensen, H. J. Aa.; Jørgensen, P.; Olsen, J.), and ECP routines (Mitin, A. V.; van Wüllen, C.); CFOUR, Coupled-Cluster Techniques for Computational Chemistry . For the current version, see http://www.cfour.de website accessed 22-Mar-2026.

[47] Dunning T. H. (1989). Gaussian basis sets for use in correlated molecular
calculations. I. The atoms boron through neon and hydrogen. J. Chem. Phys..

[48] Kendall R.
A., Dunning T. H., Harrison R. J. (1992). Electron affinities
of the first-row atoms revisited. Systematic basis sets and wave functions. J. Chem. Phys..

[49] Peterson K.
A., Dunning T. H. (2002). Accurate correlation consistent basis
sets for molecular core–valence correlation effects: The second
row atoms Al–Ar, and the first row atoms B–Ne revisited. J. Chem. Phys..

[50] Morgan W.
J., Matthews D. A., Ringholm M., Agarwal J., Gong J. Z., Ruud K., Allen W. D., Stanton J. F., Schaefer H. F. (2018). Geometric Energy Derivatives at the Complete Basis
Set Limit: Application to the Equilibrium Structure and Molecular
Force Field of Formaldehyde. J. Chem. Theory
Comput..

[51] Puzzarini C., Bloino J., Tasinato N., Barone V. (2019). Accuracy and Interpretability:
The Devil and the Holy Grail. New Routes across Old Boundaries in
Computational Spectroscopy. Chem. Rev..

[52] Gauss J., Puzzarini C. (2010). Quantum-chemical
calculation of Born-Oppenheimer breakdown
parameters to rotational constants. Mol. Phys..

[53] Puzzarini C., Stanton J. F., Gauss J. (2010). Quantum-chemical
calculation of spectroscopic
parameters for rotational spectroscopy. Int.
Rev. Phys. Chem..

[54] Corkran G., Ball D. W. (2004). The Relative Energies
of Cyclopropanone, Cyclopropanedione,
and Cyclopropanetrione. Hartree–Fock, Density-Functional, G2,
and CBS Calculations. J. Mol. Struct..

[55] Gordy, W. ; Cook, R. L. Microwave Molecular Spectra, 3rd. ed.; Wiley Interscience: New York, 1984.

[56] March, J. Advanced Organic Chemistry, 4th ed.; John Wiley & Sons: New York, 1992.

[57] Anslyn, E. V. ; Dougherty, D. A. Modern Physical Organic Chemistry; University Science Books: Sausalito, CA, 2006.

[58] Foote C. S. (1963). The Effect
of Bond Angle on Hybridization. Tetrahedron
Lett..

[59] Gauss J., Cremer D., Stanton J. F. (2000). The *r*
_e_ Structure of Cyclopropane. J. Phys. Chem.
A.

[60] Vázquez, J. ; Stanton, J. F. Semiexperimental Equilibrium Structures Computational Aspects. In Equilibrium Molecular Structures: From Spectroscopy to Quantum Chemistry; Demaison, J. , Boggs, J. E. , Császár, A. G. , Eds.; CRC Press: Boca Raton, FL, 2011; pp 53–87.

[61] Bohn R. K., Montgomery J. A., Michels H. H., Fournier J. A. (2016). Second
moments and rotational spectroscopy. J. Mol.
Spectrosc..

[62] Kraitchman J. (1953). Determination
of Molecular Structure from Microwave Spectroscopic Data. Am. J. Phys..

[63] Pochan, Baldwin, and Flygare estimated the *c* _H_ coordinate of cyclopropanone as 0.911 Å using uncorrected rotational constants (*B* _0_) and a slightly different numerical value for the unit conversion factor (505531.0 MHz amu Å^2^). See reference 8. We obtain the same value of c_H_ = 0.911 Å when using our uncorrected rotational constants and Pochan’s value for the conversion factor.

[64] Laurie V.
W. (1958). Note on
the Determination of Molecular Structure from Spectroscopic Data. J. Chem. Phys..

[65] Kuchitsu K., Bartell L. S. (1962). Effect of Anharmonic
Vibrations on the Bond Lengths
of Polyatomic Molecules. II. Cubic Constants and Equilibrium Bond
Lengths of Methane. J. Chem. Phys..

[66] Laurie V. W., Herschbach D. R. (1962). Influence of Vibrations on Molecular Structure Determinations.
II. Average Structures Derived from Spectroscopic Data. J. Chem. Phys..

[67] Morino Y., Kuchitsu K., Oka T. (1962). Internuclear
Distance Parameters. J. Chem. Phys..

[68] Duncan J. L. (1974). The ground-state
average and equilibrium structures of formaldehyde and ethylene. Mol. Phys..

[69] Cox A. P., Hubbard S. D., Kato H. (1982). The Microwave
Spectrum of Thioformaldehyde,
CD_2_S, and CH_2_S: Average Structure, Dipole Moments,
and ^33^S Quadrupole Coupling. J. Mol.
Spectrosc..

[70] Sears T. J., Frye J. M., Spirko V., Kraemer W. P. (1989). Extended measurements
of the ν_2_ band of CD_3_ and the determination
of the vibrational potential function for methyl. J. Chem. Phys..

[71] Liu J., Chen M.-W., Melnik D., Miller T. A., Endo Y., Hirota E. (2009). The spectroscopic characterization
of the methoxy radical.
II. Rotationally resolved *A*
^2^
*A*
_1_−*X*
^2^
*E* electronic and *X*
^2^
*E* microwave
spectra of the perdeuteromethoxy radical CD_3_O. J. Chem. Phys..

[72] Klaus T., Belov S. P., Winnewisser G. (1998). Precise Measurement
of the Pure Rotational
Submillimeter-Wave Spectrum of HCl and DCl in Their ν = 0, 1
States. J. Mol. Spectrosc..

[73] Isotope-dependent bond distances involving non-hydrogen atoms – CO^+ 74^ and CN,^75^ for example – differ in the sixth or seventh decimal place (in Å). Isotope-dependent bond distances involving a hydrogen atom, however, show considerably greater variability, which may reflect the difficulty of obtaining precise measurements: C–H/D (Δ = 0.000 9 Å),^76,77^ F–H/D (predicted Δ = –0.000 07 Å),^52^ Cl–H/D (Δ = –0.000 02 Å),^72^ Ga–H/D (Δ = 0.000 9 Å),^78^ Xe–H/D^+^ (Δ = –0.000 003 Å).^79^ In the majority of cases, the heavier isotopologue has the shorter bond.

[74] Spezzano S., Brünken S., Müller H. S. P., Klapper G., Lewen F., Menten K. M., Schlemmer S. (2013). Accurate High-*N* Rest
Frequencies for CO^+^, an Ideal Tracer of Photon-Dominated
Regions. J. Phys. Chem. A.

[75] Saleck A. H., Simon R., Winnewisser G. (1994). Interstellar CN Rotational Spectra: ^12^C^15^N. Astrophys. J..

[76] Bernath P. F., Brazier C. R., Olsen T., Hailey R., Fernando W. T. M. L., Woods C., Hardwick J. L. (1991). Spectroscopy of the CH Free Radical. J. Mol. Spectrosc..

[77] Wienkoop M., Urban W., Towle J. P., Brown J. M., Evenson K. M. (2003). Studies
of the mid- and far-infrared laser magnetic resonance spectra of the
CD radical: information on vibrationally excited levels. J. Mol. Spectrosc..

[78] Campbell J. M., Dulick M., Klapstein D., White J. B., Bernath P. F. (1993). High resolution
infrared emission spectra of GaH and GaD. J.
Chem. Phys..

[79] Peterson K. A., Petrmichl R. H., McClain R. L., Woods R. C. (1991). Submillimeter wave
spectroscopy of XeH^+^ and XeD^+^. J. Chem. Phys..

[80] Deakyne C.
A., Allen L. C., Laurie V. W. (1977). Bond Length Changes Resulting from
Substitution and Ring Opening in Three-Membered Systems. J. Am. Chem. Soc..

[81] Laurie V. W., Stigliani W. M. (1970). Microwave spectrum, structure, and dipole moment of
methylenecyclopropane. J. Am. Chem. Soc..

[82] Tamagawa K., Hilderbrandt R. L. (1983). Molecular structure of cyclobutanone
as determined
by combined analysis of electron diffraction and spectroscopic data. J. Phys. Chem..

[83] Tamagawa K., Hilderbrandt R., Shen Q. (1987). Molecular structure of cyclopentanone
by gas-phase electron diffraction. J. Am. Chem.
Soc..

[84] Dillen J., Geise H. (1980). The molecular structure
of cyclohexanone determined by gas-phase
electron diffraction, including microwave data. J. Mol. Struct..

[85] Turro N. J., Edelson S. S. (1968). Cyclopropanones,
X. Reaction of 2,2-Dimethylcyclopropanone
and *N*-Methylpyrrole. A New Entry Into the Tropinone
Series. J. Am. Chem. Soc..

[86] Turro N. J., Edelson S. S., Gagosian R. B. (1970). Cyclopropanones. XVIII. Cycloaddition
reactions of cyclopropanones. J. Org. Chem..

[87] Turro N. J., Edelson S. S., Williams J. R., Darling T. R., Hammond W. B. (1969). Cyclopropanones,
XII. Cycloaddition reactions of cyclopropanones. J. Am. Chem. Soc..

[88] Turro N. J., Hammond W. B. (1968). Cyclopropanones
- IX Reactions of Acids and Amines
with Cyclopropanone and Some Alkyl Cyclopropanones. Tetrahedron.

[89] Edelson S. S., Turro N. J. (1970). Cyclopropanones.
XVII. Kinetics of the Cycloaddition
Reaction of Cyclopropanones with 1,3-Dienes. J. Am. Chem. Soc..

[90] Frei K., Günthard H. H. (1961). Vibrational
Spectra and Normal Coordinate Treatment
of Cyclobutanone and α,α,α′,α′-*d*
_4_-Cyciobutanone. J. Mol.
Spectrosc..

[91] Howard-Lock H. E., King G. W. (1970). Cyclopentanone:
The vibrational spectra of some deuterated
isomers. J. Mol. Spectrosc..

[92] Galabov B., Suzuki S., Orville-Thomas W. J. (1980). Variation
of the carbonyl characteristic
frequency in cyclic ketones. Chem. Phys. Lett..

[93] Grangé, D. The Spectrum of Cyclohexanone; M.S. Dissertation; McMaster University: Hamilton, Ontario, 1971.

[94] Structure of Free Polyatomic Molecules; Kuchitsu, K. , Ed.; Springer Verlag: Berlin, 1998.

[95] Shimanouchi, T. Tables of Molecular Vibrational Frequencies; National Bureau of Standards, 1972, Vol. 39.

[96] Wiberg K. B., Nist B. J. (1961). Cyclopropene. IV.
The Infrared, Ultraviolet and N.m.r.
Spectra of Cyclopropene and Some Related Compounds. J. Am. Chem. Soc..

