# Supplementary material for: Cyclopropanone: Preparation, Rotational Spectroscopy, and Semi-Experimental Equilibrium (r e SE) Structure
Source: J Am Chem Soc. 2026 Mar 30;148(13):14072–85. doi: 10.1021/jacs.5c23052 (PMC13067281; doi:10.1021/jacs.5c23052)
Supplement: Supplementary file 1 [file ja5c23052_si_001.pdf]

Supporting Information

Cyclopropanone: Preparation, Rotational Spectroscopy, and Semi-Experimental Equilibrium (r_e^{SE}) Structure

W. Hazel Styers,[1] Brian J. Esselman,[1] Samuel A. Wood,[1]

P. Bryan Changala,[2,3,4] Gregory H. Jones,[5]

Michael C. McCarthy,[2] John F. Stanton,[5,†] R. Claude Woods,[1]* Robert J. McMahon [1]*

[1] Department of Chemistry, University of Wisconsin–Madison, Madison, Wisconsin 53706, United States

[2] Center for Astrophysics | Harvard & Smithsonian, 60 Garden Street, Cambridge, Massachusetts 02138-1516, United States

[3] JILA, University of Colorado Boulder and National Institute of Standards and Technology, Boulder, Colorado 80309, United States

[4] Department of Physics, University of Colorado Boulder, Boulder, Colorado 80309, United States

[5] Quantum Theory Project, Departments of Physics and Chemistry, University of Florida, Gainesville, Florida 32611, United States

[†] deceased March 21, 2025

* corresponding authors
E-mail address: rcwoods@wisc.edu (R.C. Woods)
E-mail address: robert.mcmahon@wisc.edu (R.J. McMahon)

Table of Contents	Page
**Eq. S1 – S6.** Calculating determinable constants	S4
**Eq. S7 – S15.** Calculating the Best Theoretical Estimate (BTE)	S5 – S6
**Table S1.** Corrections (ΔR) Used for Obtaining the BTE	S6
**Table S2.** Spectroscopic Constants of Cyclopropanone Isotopologues (S reduction, I^r representation)	S7 – S8
**Table S3.** Previously Reported Experimental Spectroscopic Constants of Cyclopropanone Isotopologues (A reduction, I^r representation)	S9
**Table S4.** Experimental and Computed Spectroscopic Constants of Cyclopropanone Isotopologues with *obs. – calc.* values	S10 – S14
**Table S5.** Determinable Spectroscopic Constants of Cyclopropanone Isotopologues	S15
**Discussion of Spectroscopic Constants**	S16
**Discussion of Second Moments**	S17
**Figures S1 – S9.** Data Distribution Plots	S18 – S26
**Synthetic Procedures and Characterization**	S27 – S39
**References**	S40 – S41

**Additional Files Provided (zipped file)**	
CCSD(T) / cc-pCVTZ geometry optimization (*cyclopropanone_freq_CCSDT_pCVTZ.out*)	Separate file
Anharmonic frequency calculations: CCSD(T), B3LYP, MP2	Separate files
CCSD(T) / cc-pCVTZ ISOMASS files – computed spectroscopic constants for cyclopropanone isotopologues (*ISOMASS##.out*)	Separate files
CCSD(T) / cc-pCVTZ magnetic calculations (*cyclopropanone_ISOMASS##_mag.out*)	Separate files
Isotopologue Fitting Files (*ISOMASS##_A_I.res, ISOMASS##_S_I.res*)	Separate files
Xrefit r_e^{SE} structure output (*cyclopropanone_reSE.out*)	Separate file
Xrefit output without computational corrections (provides $\Delta_{i\,0}$) (*cyclopropanone_r0.out*)	Separate file
Xrefit output without electron-mass distribution correction (provides $\Delta_{i\,\mathrm{e}}$ using vibration-rotation interaction correction only) (*cyclopropanone_reSE_no_e.out*)	Separate file
Xrefit r_e^{SE} structure output using alternate *z*-matrices for determination of dependent structural parameters (*cyclopropanone_reSE_alt_##.out)*	Separate file
xrefiteration output	Separate file
Additional CFOUR optimization output files used in the calculation of the Best Theoretical Estimate (BTE)	Separate files

Calculating the Determinable Constants

The ground-state rotational constants for both the S- and A-reduced Hamiltonians were converted to the "determinable constants" (A_0'', B_0'', C_0'') to account for the influence of centrifugal distortion using eq. (S1) – (S6).[1] The constants from each Hamiltonian reduction were then averaged to obtain the final determinable constants.

$$A_0'' = A_0^{(A)} + 2\Delta_J \quad \text{(S1)}$$

$$B_0'' = B_0^{(A)} + 2\Delta_J + \Delta_{JK} - 2\delta_J - 2\delta_K \quad \text{(S2)}$$

$$C_0'' = C_0^{(A)} + 2\Delta_J + \Delta_{JK} + 2\delta_J + 2\delta_K \quad \text{(S3)}$$

$$A_0'' = A_0^{(S)} + 2D_J + 6d_2 \quad \text{(S4)}$$

$$B_0'' = B_0^{(S)} + 2D_J + D_{JK} + 2d_1 + 4d_2 \quad \text{(S5)}$$

$$C_0'' = C_0^{(S)} + 2D_J + D_{JK} - 2d_1 + 4d_2 \quad \text{(S6)}$$

Ideally, the B_0'' values as determined from $B_0^{(A)}$ and $B_0^{(S)}$ are equal. The magnitude of the difference, if any, informs us as to the quality of the determinable constants. The differences between the B_0'' values for most isotopologues are well within two times the statistical uncertainties of the respective $B_0^{(A)}$ and $B_0^{(S)}$ values, and all of them are comparable in magnitude. This agreement provides confidence that both models employed in the least-squares fits are adequately treating the datasets and that the values obtained are suitable for use in the r_e^{SE} structure determination. See Table S5, below.

Calculating the Best Theoretical Estimate (BTE)

A "best theoretical estimate" (BTE) for the structure of cyclopropanone uses the CCSD(T)/cc-pCV6Z structure as a basis, with four corrections that are evaluated using conventional methodology:[2-3]

1. Residual basis-set effects beyond cc-pCV6Z: To estimate the correction needed to approach the infinite basis set limit, equilibrium structural parameters obtained with the cc-pCVXZ (X = Q, 5, 6) basis sets were extrapolated using the empirical exponential expression in Eq. (S7).[2-4]

$$R(x) = R(\infty) + Ae^{-Bx} \tag{S7}$$

R(x) are the values of the parameters obtained using the various basis sets (x = 4, 5, and 6), and R(∞) is the desired basis set limit estimate. Using three basis sets (x = 4, 5, and 6), the system of equations using Eq. (S7) can be solved, yielding Eq. (S8).[2]

$$R(\infty) = -\frac{R(5)^2 - R(4)R(6)}{R(4)+R(6)-2R(5)} \tag{S8}$$

An alternate two-point extrapolation can be determined with Eq. S9 and Eq. S10.[5-8]

$$R(x) = R(\infty) + A\left(x+\frac{1}{2}\right)^{-4} \tag{S9}$$

$$R(\infty) = \frac{6.5^4 R(6) - 5.5^4 R(5)}{6.5^4 - 5.5^4} \tag{S10}$$

Three-point extrapolation (S8) was used for all parameters except $\theta_{C2–C1–O}$. Two-point extrapolation (S10) was used for $\theta_{C2–C1–O}$ because the computed values for this parameter displayed anomalous convergence behavior for the series cc-pCVXZ, X = Q, 5, 6.

The correction to the structure due to a finite basis set is estimated by Eq. (S11).

$$\Delta R(\text{basis}) = R(\infty) - R(\text{CCSD(T)/cc-pCV6Z}) \tag{S11}$$

2. Residual electron correlation effects beyond the CCSD(T) treatment: Residual correlation effects are assessed by doing geometry optimizations at the CCSDT(Q) level[9] and then estimating the correlation correction in Eq. (S12).

$$\Delta R(\text{cor}) = R(\text{CCSDT(Q)}) - R(\text{CCSD(T)}) \tag{S12}$$

Calculations at the CCSDT(Q) level of theory are computationally demanding. In previous studies, we performed these calculations with the cc-pVDZ basis in the frozen-core approximation.[10-11] In the current study, we performed these calculations with the cc-pVTZ basis in the frozen-core approximation.

3. Effects of scalar (mass-velocity and Darwin) relativistic effects: The relativistic corrections are obtained by subtraction of the equilibrium parameters obtained with a standard non-relativistic calculation from those obtained with the X2C-1e variant of coupled-cluster theory,[12-14] as shown in Eq. (S13).

$$\Delta R(\text{rel}) = R(\text{CCSD(T)/XC2-pCVTZ})_{\text{SFX2C-1e}} - R(\text{CCSD(T)/cc-pCVTZ})_{NR} \quad \text{(S13)}$$

The relativistic calculation was performed with the re-contracted basis set XC2-pCVTZ, the non-relativistic calculation was performed with the cc-pVTZ basis set. All electrons were correlated in both calculations. (Keyword: RELATIVISTIC=X2C1E)

4. The diagonal Born-Oppenheimer correction (DBOC): The diagonal Born-Oppenheimer correction (DBOC)[15-16] is obtained from Eq. (S14).

$$\Delta R(\text{DBOC}) = R(\text{SCF/cc-pVTZ})_{DBOC} - R(\text{SCF/cc-pVTZ})_{NR} \quad \text{(S14)}$$

Here, the first value is obtained by minimizing the DBOC-corrected SCF energy with respect to nuclear positions, and the latter is again the traditional calculation. (Keyword: DBOC=ON)

(For more detail concerning corrections for non-Born-Oppenheimer behavior, see the following references: 2, 17-18)

The sum of the above-described corrections is used to obtain the best equilibrium structural parameters, given by Eq. (S15).

$$\Delta R(\text{total}) = \Delta R(\text{basis}) + \Delta R(\text{cor}) + \Delta R(\text{rel}) + \Delta R(\text{DBOC}) \quad \text{(S15)}$$

The sum of the corrections, ΔR(total), is applied to the CCSD(T)/cc-pCV6Z structural parameters.

Table S1. Corrections (ΔR) Applied to Computed Structural Parameters (CCSD(T)/cc-pCV6Z) to Afford the Best Theoretical Estimate (BTE)

	ΔR (basis)	ΔR (corr)	ΔR (rel)	ΔR (DBOC)	ΔR (total)
$R_{\text{C1–O}}$	−0.000 27	0.000 94	−0.000 11	0.000 05	0.000 61
$R_{\text{C1–C2}}$	−0.000 14	0.000 53	−0.000 15	0.000 00	0.000 24
$R_{\text{C2–H}}$	0.000 00	0.000 13	−0.000 11	0.000 13	0.000 15
$\theta_{\text{C2–C1–O}}$	0.001 83	−0.003 05	−0.003 17	−0.002 54	−0.006 93
$\theta_{\text{C1–C2–H}}$	−0.006 11	0.010 07	0.001 00	−0.002 54	0.002 42
$\theta_{\text{H–C2–C1-O}}$	−0.011 69	0.000 81	0.006 99	0.001 70	−0.002 19

Distance (R) in Ångstrom (Å); angle (θ) in degree (°).

Table S2. Spectroscopic Constants of Cyclopropanone Isotopologues (S reduction, I^r representation) [a]

	normal isotopologue	[1-^{13}C]	[2-^{13}C]	[^{18}O]
A_0 (MHz)	20153.3672(30)	20152.55(19)	19682.2192(84)	20153.322(79)
B_0 (MHz)	7466.60986(14)	7457.51865(83)	7370.71287(47)	7017.37636(32)
C_0 (MHz)	5871.88315(12)	5866.2871(10)	5772.38481(39)	5590.32576(32)
D_J (kHz)	1.609915(41)	1.539(43)	1.561439(94)	1.367(35)
D_{JK} (kHz)	18.81121(25)	19.8(10)	18.18064(83)	[16.9532]
D_K (kHz)	20.620(22)	[19.701]	[18.8102]	[21.1992]
d_1 (kHz)	−0.443811(39)	−0.402(20)	−0.438804(99)	[−0.3529]
d_2 (kHz)	−0.135846(29)	−0.1302(32)	−0.136954(75)	[−0.1024]
H_J (Hz)	0.0004905(77)	[0.0004656]	[0.0004318]	[0.000372]
H_{JK} (Hz)	−0.005126(49)	[−0.001215]	[−0.002159]	[0.001243]
H_{KJ} (Hz)	0.23474(23)	[0.2055]	[0.2012]	[0.1830]
H_K (Hz)	[−0.2530]	[−0.2511]	[−0.2439]	[−0.2310]
h_1 (Hz)	0.0002560(77)	[0.0002345]	[0.0002165]	[0.0001966]
h_2 (Hz)	0.0001474(72)	[0.0001157]	[0.0001175]	[0.0000969]
h_3 (Hz)	0.0001382(29)	[0.0001097]	[0.0001134]	[0.0000819]
κ [b]	−0.776 7	−0.777 2	−0.770 2	−0.804 0
$P_{cc\ 0}$ (uÅ^2) [c]	3.347 119 (2)	3.347 83 (12)	3.345 797 (7)	3.346 26 (5)
N_{lines} [d]	1390 [e]	30 [e]	263 [e]	5
σ (MHz)	0.034	0.068	0.054	0.0002

[a] Value in brackets held fixed at the CCSD(T)/cc-pCVTZ value.
[b] Calculated using PLANM from the B_0 constants.
[c] $P_{cc} = -(I_c - I_a - I_b)/2 = -½\,\Delta_{i\,0}$, calculated using PLANM
[d] Number of independent transitions.
[e] Includes transitions reported by Pochan *et al.*[19]

Table S2 *cont*. Spectroscopic Constants of Cyclopropanone Isotopologues (S reduction, I^r representation) [a]

	[2-^{2}H]	[2,2-^{2}H]	*trans*-[2,3-^{2}H]	*cis*-[2,3-^{2}H]	[2,2,3-^{2}H]	[2,2,3,3-^{2}H]
A_0 (MHz)	18412.8593(36)	17000.0102(64)	16808.4184(41)	16887.0044(40)	15575.7580(32)	14415.9449(47)
B_0 (MHz)	7216.19663(24)	6960.84572(32)	7009.92870(33)	6988.28844(34)	6773.40355(29)	6574.49867(29)
C_0 (MHz)	5659.31199(21)	5478.09498(27)	5455.11859(28)	5467.09068(27)	5291.66784(43)	5132.23726(26)
D_J (kHz)	1.540775(41)	1.470694(80)	1.412222(56)	1.463746(57)	1.35245(25)	1.25818(13)
D_{JK} (kHz)	17.29199(53)	15.32549(71)	17.02441(28)	15.80799(27)	14.89313(18)	13.75329(66)
D_K (kHz)	[14.0757]	10.436(45)	[7.23351]	[10.5985]	[5.37599]	2.472(24)
d_1 (kHz)	−0.419323(43)	−0.390206(49)	−0.387846(56)	−0.390403(56)	−0.359140(49)	−0.330405(44)
d_2 (kHz)	−0.123979(30)	−0.093895(32)	−0.127981(34)	−0.112516(33)	−0.097555(26)	−0.082913(24)
H_J (Hz)	[0.0005032]	[0.000464]	[0.0002733]	[0.0007591]	0.000360(71)	0.000356(38)
H_{JK} (Hz)	[0.008252]	0.01292(29)	[0.01834]	[0.006269]	[0.01462]	0.01371(25)
H_{KJ} (Hz)	0.16054(83)	0.09955(97)	[0.09583]	[0.1259]	[0.07845]	0.07135(53)
H_K (Hz)	[−0.1674]	[−0.1022]	[−0.1245]	[−0.1385]	[−0.09500]	[−0.07795]
h_1 (Hz)	[0.0002548]	[0.0002289]	[0.0001823]	[0.0003138]	[0.0002182]	[0.0001974]
h_2 (Hz)	[0.0001359]	[0.0000724]	[0.0001859]	[0.0000974]	[0.0000797]	[0.0000295]
h_3 (Hz)	[0.0000984]	[0.000052]	[0.0001053]	[0.0000878]	[0.0000616]	[0.0000426]
κ [b]	−0.755 9	−0.742 6	−0.726 1	−0.733 6	−0.711 8	−0.689 3
$P_{cc\ 0}$ (uÅ^2) [c]	4.090 311 (4)	5.038 373 (7)	4.759 339 (5)	4.902 441 (5)	5.777 054 (6)	6.727 527 (7)
N_{lines} [d]	634	658 [e]	491	491	624	626
σ (MHz)	0.035	0.060	0.045	0.045	0.042	0.045

[a] Value in brackets held fixed at the CCSD(T)/cc-pCVTZ value.

[b] Calculated using PLANM from the B_0 constants.

[c] $P_{cc} = -(I_c - I_a - I_b)/2 = -½\, \Delta_{i\,0}$, calculated using PLANM

[d] Number of independent transitions.

[e] Includes transitions reported by Pochan *et al.*[19]

Table S3. Previously Reported Experimental Spectroscopic Constants of Cyclopropanone Isotopologues (A reduction, I^r representation) [a]

	normal isotopologue	[1-^{13}C]	[2-^{13}C]	[2,2-^{2}H]
A_0 (MHz)	20155.11 (39)	20155 (10)	19684.93 (465)	17004.98 (600)
B_0 (MHz)	7466.52 (4)	7457.3 (3)	7370.60 (5)	6960.80 (11)
C_0 (MHz)	5871.85 (4)	5866.2 (2)	5772.29 (4)	5477.91 (9)
κ [b]	−0.776 7	−0.7773	−0.7702	−0.7427
$P_{cc\ 0}$ (uÅ^2) [c]	3.346 20 (42)	3.347 (7)	3.3439 (31)	5.033 (5)
N_{lines} [d]	15	4	6	7

[a] Spectroscopic constants from Pochan *et al.*[19]

[b] Calculated using PLANM from the B_0 constants.

[c] $P_{cc} = -(I_c - I_a - I_b)/2 = -½\ \Delta_{i\ 0}$, calculated using PLANM

[d] Number of independent transitions reported by Pochan *et al.*[19]

Table S4. Experimental and Computed Spectroscopic Constants of Cyclopropanone Isotopologues (A reduction, I^r representation), with *obs. – calc.* values [a]

	normal isotopologue			[1-^{13}C]		
	Experimental	CCSD(T)[b]	*obs. – calc.*	Experimental	CCSD(T)[b]	*obs. – calc.*
A_0 (MHz)	20153.365 8 (30)	20073.237 9	80.1279	20152.655 (76)	20072.577 8	80.0772
B_0 (MHz)	7466.628 73 (14)	7419.292 3	47.3365	7457.536 42 (71)	7410.164 6	47.3718
C_0 (MHz)	5871.865 29 (12)	5836.328 6	35.5367	5866.269 76 (65)	5830.710 1	35.5596
Δ_J (kHz)	1.881 587 (70)	1.790 7	0.0910	1.821 64 (28)	1.793 3	0.0284
Δ_{JK} (kHz)	17.180 41 (39)	16.952 6	0.2278	17.612 (25)	16.793 1	0.819
Δ_K (kHz)	21.992 (22)	20.791 0	1.201	[20.948]	20.948 0	
δ_J (kHz)	0.443 817 (39)	0.413 1	0.0307	[0.413 759]	0.413 8	
δ_K (kHz)	9.191 5 (20)	8.766 8	0.4247	[8.728 26]	8.728 3	
Φ_J (Hz)	0.000 786 (17)	0.000 694 5	0.000092	[0.000 697]	0.000 697 0	
Φ_{JK} (Hz)	0.029 15 (74)	0.028 82	0.00033	[0.028 105 5]	0.028 11	
Φ_{KJ} (Hz)	0.114 5 (26)	0.104 8	0.0097	[0.104 285 8]	0.104 3	
Φ_K (Hz)	[−0.180 7]	−0.180 7		[−0.179 5]	−0.179 5	
ϕ_J (Hz)	0.000 394 3 (88)	0.000 342 3	0.0000520	[0.000 344 2]	0.000 344 2	
ϕ_{JK} (Hz)	0.010 47 (47)	0.009 317	0.00115	[0.009 114 3]	0.009 114	
ϕ_K (Hz)	0.336 8 (90)	0.288 6	0.0482	[0.287 522 8]	0.287 5	
κ [c]	−0.776 7	−0.778		−0.777 2	−0.778	
$P_{cc\ 0}$ (uÅ^2) [d]	3.346 903 (2)	3.350		3.347 559 (48)	3.352	
N_{lines} [e]	1390[f]			30[f]		
σ (MHz)	0.035			0.069		

[a] Values in square brackets held fixed at the CCSD(T)/cc-pCVTZ predicted value in the least-squares fit. [b] B_0 values and distortion constants computed using CCSD(T)/cc-pCVTZ. [c] Calculated using PLANM from the B_0 constants. [d] $P_{cc} = -(I_c - I_a - I_b) / 2 = -½\, \Delta_{i\ 0}$, calculated using PLANM [e] Number of independent transitions. [f] Includes transitions reported by Pochan *et al.* [19]

Table S4 *cont.* Experimental and Computed Spectroscopic Constants of Cyclopropanone Isotopologues (A reduction, I^r representation), with *obs. – calc.* values [a]

	[2-^{13}C]			[^{18}O]		
	Experimental	CCSD(T)[b]	*obs. – calc.*	Experimental	CCSD(T)[b]	*obs. – calc.*
A_0 (MHz)	19682.209 5 (84)	19603.334 8	78.8747	20153.302 (79)	20073.092 3	80.2097
B_0 (MHz)	7370.731 17 (47)	7323.994 3	46.7369	7017.393 17 (32)	6973.029 0	44.3642
C_0 (MHz)	5772.367 49 (39)	5737.438 6	34.9289	5590.309 77 (32)	5556.488 2	33.8215
Δ_J (kHz)	1.835 32 (19)	1.746 2	0.0891	1.571 (35)	1.586 7	−0.0157
Δ_{JK} (kHz)	16.536 3 (11)	16.326 6	0.2097	[15.724 7]	15.724 7	
Δ_K (kHz)	[20.079 9]	20.079 9		[22.222 9]	22.222 9	
δ_J (kHz)	0.438 786 (99)	0.407 9	0.0309	[0.352 88]	0.352 9	
δ_K (kHz)	8.988 7 (49)	8.593 4	0.3953	[8.200 37]	8.200 4	
Φ_J (Hz)	[0.000 666 7]	0.000 666 7		[0.000 565 7]	0.000 565 7	
Φ_{JK} (Hz)	[0.027 125 3]	0.027 13		[0.026 326 8]	0.026 33	
Φ_{KJ} (Hz)	[0.100 068 8]	0.100 1		[0.096 508]	0.096 51	
Φ_K (Hz)	[−0.172 3]	−0.172 3		[−0.169 8]	−0.169 8	
ϕ_J (Hz)	[0.000 329 9]	0.000 329 9		[0.000 278 5]	0.000 278 5	
ϕ_{JK} (Hz)	[0.009 104]	0.009 104		[0.008 349 6]	0.008 350	
ϕ_K (Hz)	[0.278 198 4]	0.278 2		[0.280 419]	0.280 4	
κ [c]	−0.770 2	−0.771 2		−0.804 0	−0.804 8	
$P_{cc\ 0}$ (uÅ^2) [d]	3.345 586 (7)	3.349 5		3.346 053 (49)	3.350 5	
N_{lines} [e]	263 [f]			5		
σ (MHz)	0.053			0.000 22		

[a] Values in square brackets held fixed at the CCSD(T)/cc-pCVTZ predicted value in the least-squares fit. [b] B_0 values and distortion constants computed using CCSD(T)/cc-pCVTZ. [c] Calculated using PLANM from the B_0 constants. [d] $P_{cc} = -(I_c - I_a - I_b) / 2 = -½ \Delta_{i\ 0}$, calculated using PLANM [e] Number of independent transitions. [f] Includes transitions reported by Pochan *et al.* [19]

Table S4 *cont.* Experimental and Computed Spectroscopic Constants of Cyclopropanone Isotopologues (A reduction, I^r representation), with *obs. – calc.* values [a]

	[2-^{2}H]			[2,2-^{2}H]		
	Experimental	CCSD(T)[b]	*obs. – calc.*	Experimental	CCSD(T)[b]	*obs. – calc.*
A_0 (MHz)	18412.848 2 (37)	18341.831 2	71.0170	17000.008 2 (64)	16935.989 3	64.0189
B_0 (MHz)	7216.211 81 (23)	7170.474 3	45.7375	6960.856 95 (31)	6916.919 9	43.9370
C_0 (MHz)	5659.297 39 (21)	5625.066 1	34.2313	5478.084 46 (27)	5444.943 9	33.1406
Δ_J (kHz)	1.788 633 (73)	1.705 0	0.0836	1.658 472 (89)	1.584 3	0.0742
Δ_{JK} (kHz)	15.802 80 (57)	15.528 5	0.2743	14.198 67 (72)	13.907 8	0.2909
Δ_K (kHz)	[15.228 1]	15.228 1		11.363 (45)	10.772 0	0.591
δ_J (kHz)	0.419 264 (43)	0.391 3	0.0280	0.390 198 (49)	0.365 6	0.0246
δ_K (kHz)	7.629 2 (18)	7.302 5	0.3268	5.461 9 (19)	5.209 9	0.2520
Φ_J (Hz)	[0.000 774 9]	0.000 774 9		[0.000 608 7]	0.000 608 7	
Φ_{JK} (Hz)	[0.031 570 4]	0.031 57		0.024 91 (29)	0.025 41	−0.00050
Φ_{KJ} (Hz)	0.076 45 (82)	0.060 61	0.01584	0.057 11 (97)	0.048 38	0.00872
Φ_K (Hz)	[−0.109 2]	−0.109 2		[−0.073 14]	−0.073 14	
ϕ_J (Hz)	[0.000 353 2]	0.000 353 2		[0.000 280 9]	0.000 280 9	
ϕ_{JK} (Hz)	[0.010 07]	0.010 07		[0.005 561]	0.005 561	
ϕ_K (Hz)	[0.212 2]	0.212 2		[0.097 10]	0.097 10	
κ [c]	−0.755 8	−0.757 0		−0.742 6	−0.743 8	
$P_{cc\ 0}$ (uÅ^2) [d]	4.090 130 (4)	4.095 3		5.038 228 (7)	5.044 5	
N_{lines} [e]	634			658[f]		
σ (MHz)	0.035			0.060		

[a] Values in square brackets held fixed at the CCSD(T)/cc-pCVTZ predicted value in the least-squares fit. [b] B_0 values and distortion constants computed using CCSD(T)/cc-pCVTZ. [c] Calculated using PLANM from the B_0 constants. [d] $P_{cc} = -(I_c - I_a - I_b) / 2 = -½\ \Delta_{i\ 0}$, calculated using PLANM [e] Number of independent transitions. [f] Includes transitions reported by Pochan *et al.* [19]

Table S4 *cont.* Experimental and Computed Spectroscopic Constants of Cyclopropanone Isotopologues (A reduction, I^r representation), with *obs. – calc.* values [a]

	trans-[2,3-^{2}H]			*cis*-[2,3-^{2}H]		
	Experimental	CCSD(T)[b]	*obs. – calc.*	Experimental	CCSD(T)[b]	*obs. – calc.*
A_0 (MHz)	16808.405 6 (41)	16745.175 6	63.2300	16886.993 7 (41)	16823.417 8	63.5759
B_0 (MHz)	7009.942 62 (33)	6965.420 7	44.5219	6988.301 08 (34)	6944.078 0	44.2230
C_0 (MHz)	5455.105 42 (28)	5422.195 0	32.9104	5467.078 71 (27)	5433.994 7	33.0840
Δ_J (kHz)	1.668 140 (90)	1.593 2	0.0750	1.688 737 (91)	1.612 0	0.0767
Δ_{JK} (kHz)	15.487 97 (46)	15.149 9	0.3381	14.457 35 (44)	14.173 4	0.2840
Δ_K (kHz)	[8.427 3]	8.427 3		[11.649 9]	11.649 9	
δ_J (kHz)	0.387 805 (56)	0.362 5	0.0253	0.390 365 (56)	0.365 3	0.0250
δ_K (kHz)	6.964 8 (18)	6.680 5	0.2843	6.307 9 (18)	6.059 6	0.2483
Φ_J (Hz)	[0.000 6451]	0.000 645 1		[0.000 953 8]	0.000 953 8	
Φ_{JK} (Hz)	[0.039 68]	0.039 68		[0.025 34]	0.025 34	
Φ_{KJ} (Hz)	[0.019 13]	0.019 13		[0.059 44]	0.059 44	
Φ_K (Hz)	[−0.069 48]	−0.069 48		[−0.091 26]	−0.091 26	
ϕ_J (Hz)	[0.000 287 6]	0.000 287 6		[0.000 401 6]	0.000 401 6	
ϕ_{JK} (Hz)	[0.013 07]	0.013 07		[0.007 254]	0.007 254	
ϕ_K (Hz)	[0.172 1]	0.172 1		[0.158 5]	0.158 5	
κ [c]	−0.726 1	−0.727 4		−0.733 6	−0.734 8	
$P_{cc\ 0}$ (uÅ^2) [d]	4.759 166 (5)	4.770 5		4.902 284 (5)	4.908 0	
N_{lines} [e]	491			491		
σ (MHz)	0.045			0.045		

[a] Values in square brackets held fixed at the CCSD(T)/cc-pCVTZ predicted value in the least-squares fit. [b] B_0 values and distortion constants computed using CCSD(T)/cc-pCVTZ. [c] Calculated using PLANM from the B_0 constants. [d] $P_{cc} = -(I_c - I_a - I_b) / 2 = -½ \Delta_{i\ 0}$, calculated using PLANM [e] Number of independent transitions. [f] Includes transitions reported by Pochan *et al.* [19]

Table S4 *cont.* Experimental and Computed Spectroscopic Constants of Cyclopropanone Isotopologues (A reduction, I^r representation), with *obs. – calc.* values [a]

	[2,2,3-^{2}H]			[2,2,3,3-^{2}H]		
	Experimental	CCSD(T)[b]	*obs. – calc.*	Experimental	CCSD(T)[b]	*obs. – calc.*
A_0 (MHz)	15575.747 3 (32)	15518.361 4	57.3859	14415.943 4 (47)	14363.782 1	52.1613
B_0 (MHz)	6773.413 65 (29)	6730.625 0	42.7887	6574.506 83 (29)	6533.096 3	41.4106
C_0 (MHz)	5291.658 57 (43)	5259.691 6	31.9669	5132.229 75 (26)	5101.241 6	30.9882
Δ_J (kHz)	1.547 64 (26)	1.479 9	0.0677	1.423 99 (13)	1.363 2	0.0608
Δ_{JK} (kHz)	13.722 09 (34)	13.393 9	0.3282	12.758 40 (67)	12.409 1	0.3493
Δ_K (kHz)	[6.287 9]	6.287 9		3.293 (24)	3.220 2	0.0728
δ_J (kHz)	0.359 100 (49)	0.336 9	0.0222	0.330 397 (44)	0.310 7	0.0197
δ_K (kHz)	5.026 2 (14)	4.825 6	0.2006	3.937 7 (12)	3.783 6	0.1541
Φ_J (Hz)	0.000 549 (71)	0.000 644 0	−0.000095	0.000 411 (38)	0.000 542 3	−0.000131
Φ_{JK} (Hz)	[0.026 71]	0.026 71		0.022 00 (25)	0.022 42	−0.00042
Φ_{KJ} (Hz)	[0.035 77]	0.035 77		0.042 78 (52)	0.034 49	0.00829
Φ_K (Hz)	[−0.064 57]	−0.064 57		[−0.058 56]	−0.058 56	
ϕ_J (Hz)	[0.000 279 9]	0.000 279 9		[0.000 24]	0.000 240 0	
ϕ_{JK} (Hz)	[0.006 207]	0.006 207		[0.003 394]	0.003 394	
ϕ_K (Hz)	[0.089 41]	0.089 41		[0.050 14]	0.050 14	
κ [c]	−0.711 8	−0.713 2		−0.689 3	−0.690 8	
$P_{cc\ 0}$ (uÅ^2) [d]	5.776 925 (6)	5.784 1		6.727 409 (7)	6.735 9	
N_{lines} [e]	624			626		
σ (MHz)	0.042			0.045		

[a] Values in square brackets held fixed at the CCSD(T)/cc-pCVTZ predicted value in the least-squares fit. [b] B_0 values and distortion constants computed using CCSD(T)/cc-pCVTZ. [c] Calculated using PLANM from the B_0 constants. [d] $P_{cc} = -(I_c - I_a - I_b) / 2 = -½ \Delta_{i\ 0}$, calculated using PLANM [e] Number of independent transitions. [f] Includes transitions reported by Pochan *et al.* [19]

Table S5. Determinable Spectroscopic Constants of Cyclopropanone Isotopologues

	normal isotopologue	[1-^{13}C]	[2-^{13}C]	[1-^{18}O]
$A''^{(A)}$ (MHz)	20153.36956	20152.65864	19682.21317	20153.30514
$B''^{(A)}$ (MHz)	7466.630403	7457.539391	7370.732522	7017.39493
$C''^{(A)}$ (MHz)	5871.905504	5866.309299	5772.406552	5590.345743
$A''^{(S)}$ (MHz)	20153.369605	20152.55230	19682.22150	20153.32412
$B''^{(S)}$ (MHz)	7466.630460	7457.54020	7370.73275	7017.39493
$C''^{(S)}$ (MHz)	5871.905525	5866.31026	5772.40644	5590.34574
$A''^{(Avg)}$ (MHz)	20153.369584	20152.61	19682.2173	20153.315
$B''^{(Avg)}$ (MHz)	7466.630431	7457.53980	7370.73264	7017.3949311
$C''^{(Avg)}$ (MHz)	5871.905515	5866.30978	5772.40650	5590.34574333

	[2-^{2}H]	[2,2-^{2}H]	*trans*-[2,3-^{2}H]	*cis*-[2,3-^{2}H]	[2,2,3-^{2}H]	[2,2,3,3-^{2}H]
$A''^{(A)}$ (MHz)	18412.85178	17000.01152	16808.40894	16886.99708	15575.7504	14415.94625
$B''^{(A)}$ (MHz)	7216.215093	6960.862761	7009.946739	6988.305518	6773.419697	6574.5139
$C''^{(A)}$ (MHz)	5659.332867	5478.11368	5455.138949	5467.109941	5291.686158	5132.253893
$A''^{(S)}$ (MHz)	18412.86164	17000.01258	16808.42046	16887.00665	15575.76012	14415.94692
$B''^{(S)}$ (MHz)	7216.21567	6960.86283	7009.94726	6988.30594	6773.42004	6574.51395
$C''^{(S)}$ (MHz)	5659.33271	5478.11365	5455.13870	5467.10975	5291.68577	5132.25386
$A''^{(Avg)}$ (MHz)	18412.8567	17000.0120	16808.415	16887.0019	15575.7553	14415.94658
$B''^{(Avg)}$ (MHz)	7216.21538	6960.862796	7009.94700	6988.30573	6773.41987	6574.513924
$C''^{(Avg)}$ (MHz)	5659.33279	5478.113666	5455.13883	5467.10984	5291.68596	5132.253876

Discussion of Spectroscopic Constants

The experimental and computed rotational and centrifugal distortion constants (CCSD(T)/cc-pCVTZ) show excellent agreement (Table S4 of Supporting Information). All rotational constants agree within 0.64%, all determined on-diagonal quartic centrifugal distortion terms agree within 5.5%, and all determined off-diagonal terms agree within 7.5%. This is consistent with similar work on ketene,[11] giving further confidence that these parameters are well determined experimentally and physically meaningful in both works. Despite this good agreement, a systematic underprediction of the spectroscopic constants was observed, highlighting an area in which theory may be improved. As expected, the spectroscopic constants were underpredicted for all isotopologues and the consistency of the discrepancies between the computed values and their experimental values was able to be exploited for spectral predictions. For each isotopologue, the computed rotational constants were consistently underpredicted by 30-80 MHz, relative to the experimental values. Similarly, the quartic centrifugal distortion constants are consistently underpredicted. The [1-^{13}C] and [^{18}O]-isotopologue data sets have fewer measured transitions and smaller J and K_a ranges, which reduces the reliability of the distortion-constant determination. Excluding those two isotopologues, all the quartic centrifugal distortion constants were underpredicted: Δ_J (0.061 kHz to 0.091 kHz), Δ_{JK} (0.021 kHz to 0.035 kHz), δ_J (0.020 kHz to 0.031 kHz), and δ_K (0.15 to 0.40 kHz). The value of Δ_K was not sufficiently well determined for enough isotopologues to provide a similar analysis, due in large part to the availability of only a small number of a-type transitions for many species. Of the determined sextic constants, the level of agreement is expectedly poorer, with discrepancies between theory and experiment for these terms being as high as 32%. Very few sextic constants could be determined (Tables 1 and S2). Analysis of the differences between computed and experimental sextic centrifugal distortion constants is not reliable, as a complete set of sextic centrifugal distortion constants was not determined for most of the isotopologues. The *obs.* − *calc.* values for the spectroscopic constants are included in Table S4.

Discussion of Second Moments

The inertial defects ($\Delta_i = -2P_{cc}$) or second-moments (P_{bb} or P_{cc}) provide information about the quality of the equilibrium rotational constants that result from applying computed corrections to the experimental determinable constants. Cyclopropanone has four hydrogen atoms that lie outside of the *ab*-plane, resulting in a distinctly non-zero inertial defect and P_{cc}. These are the only out-of-plane atoms, thus isotopologues that have the same isotopic substitution of these atoms, e.g., [1-^{13}C]-, [2-^{13}C]-, [^{18}O]-, and cyclopropanone, should have the same P_{cc} (and Δ_i) values. The second moments for all isotopologues are provided in the main article as Table 1 (uncorrected, $P_{cc\,0}$) and Table 4 (semi-experimental, $P_{cc\,e}$,with both vibration-rotation and electron mass corrections). The variation in P_{cc} values between [1-^{13}C]-, [2-^{13}C]-, [^{18}O]-, and the normal isotopologue of cyclopropanone decreases upon correction for the vibration-rotation interaction and electron-mass contribution. Before correction, the standard deviation of those $P_{cc\,0}$ values is 0.000 88 uÅ^2, while after correction, the standard deviation of those $P_{cc\,e}$ values is 0.000 08 uÅ^2. The $P_{cc\,e}$ value of the [2,2,3,3-^{2}H]-isotopologue is, as expected, almost exactly twice that of each of the all-protio isotopologues due to the out-of-plane ^{2}H atoms. Similar to P_{cc} for isotopologues with identical isotopic substitution in the *ab* plane, P_{bb} is ideally the same for all isotopologues with identical isotopic substitution in the *ac* plane, *e.g.*, [1-^{13}C]- and [^{18}O]-cyclopropanone. The standard deviation in $P_{bb\,0}$ for [1-^{13}C]-, [^{18}O]-, and the normal isotopologue of cyclopropanone is 0.000 48 uÅ^2, which decreases to only 0.000 06 uÅ^2 for $P_{bb\,e}$ after the corrections are applied. Collectively, these analyses provide high confidence in the experimental rotational constants and their computed corrections used to determine the r_e^{SE} structure.

(Table 4 reproduced from original article)

Second Moments ($P_{bb\,e}$)^a of Cyclopropanone Isotopologues with Vibration-Rotation and Electron-Mass Corrections

Isotopologue	$P_{aa\,e}$ (uÅ^2)	$P_{bb\,e}$ (uÅ^2)	$P_{cc\,e}$ (uÅ^2)	c_H (Å) ^{b}
C_3H_4O	64.1403	21.4023	3.365 31	0.913 671
[1-^{13}C]	64.2281	21.4024	3.365 34	0.913 675
[2-^{13}C]	65.0205	21.9980	3.365 31	0.913 671
[^{18}O]	68.4671	21.4023	3.365 16	0.913 650
[2-^{2}H]	65.7319	23.0271	4.103 64	
[2,2-^{2}H]	67.3345	24.3581	5.046 50	
trans-[2,3-^{2}H]	67.1130	24.9709	4.768 85	
cis-[2,3-^{2}H]	67.1905	24.6931	4.910 94	
[2,2,3-^{2}H]	68.5950	26.3309	5.781 37	
[2,2,3,3-^{2}H]	69.8874	27.9840	6.727 59	0.913 817

^{a} $P_{bb} = -(I_b - I_a - I_c)/2$, where P_{aa} and P_{cc} can be determined by the appropriate permutation of this equation. Second moments calculated using I_e values, which were obtained by applying corrections for vibration-rotation interaction and electron mass distribution to I_0 values. ^{b} *c*-coordinate of the H / D atom; $c_H = (P_{cc}/4m_H)^{1/2}$ = distance from the *a,b*-plane (heavy-atom plane); m_H = 1.007825035 amu for ^{1}H, 2.014101779 amu for ^{2}H.

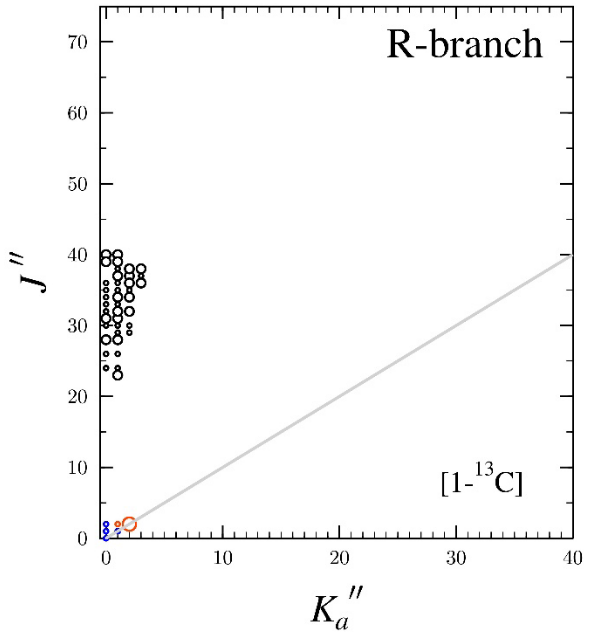

Figure S1 - Data distribution plot for the least-squares fit of spectroscopic data for the vibrational ground state of [1-^{13}C]-cyclopropanone. The size of the symbol is proportional to the value of $|(f_{obs.} - f_{calc.})/\delta f|$, where δf is the frequency measurement uncertainty of the relevant transition, and all quotient values are smaller than 3. Black circles represent millimeter-wave data, blue symbols represent microwave transitions recorded by the FT-microwave spectrometer, and orange symbols represent microwave data from Pochan *et al.*[19]

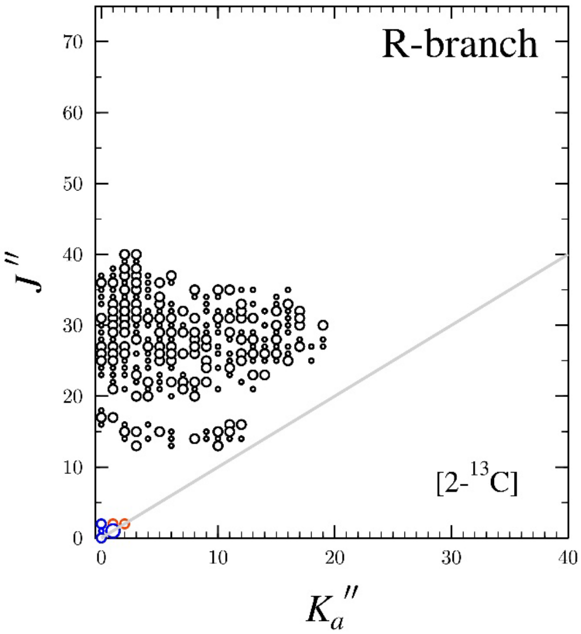

Figure S2 - Data distribution plot for the least-squares fit of spectroscopic data for the vibrational ground state of [2-^{13}C]-cyclopropanone. The size of the symbol is proportional to the value of $|(f_{obs.} - f_{calc.})/\delta f|$, where δf is the frequency measurement uncertainty of the relevant transition, and all quotient values are smaller than 3. Black circles represent millimeter-wave data, blue symbols represent microwave transitions recorded by the FT-microwave spectrometer, and orange symbols represent microwave data from Pochan *et al.*[19]

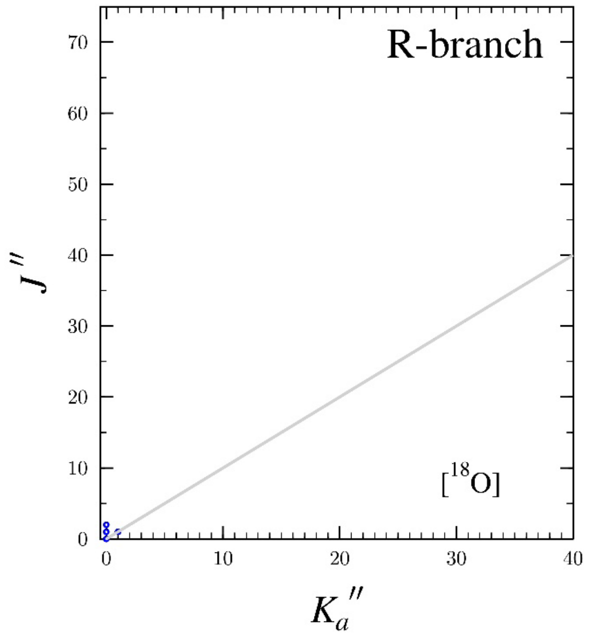

Figure S3 - Data distribution plot for the least-squares fit of spectroscopic data for the vibrational ground state of [^{18}O]-cyclopropanone. The size of the symbol is proportional to the value of $|(f_{obs.} - f_{calc.})/\delta f|$, where δf is the frequency measurement uncertainty of the relevant transition, and all quotient values are smaller than 3. Blue symbols represent microwave transitions recorded by the FT-microwave spectrometer.

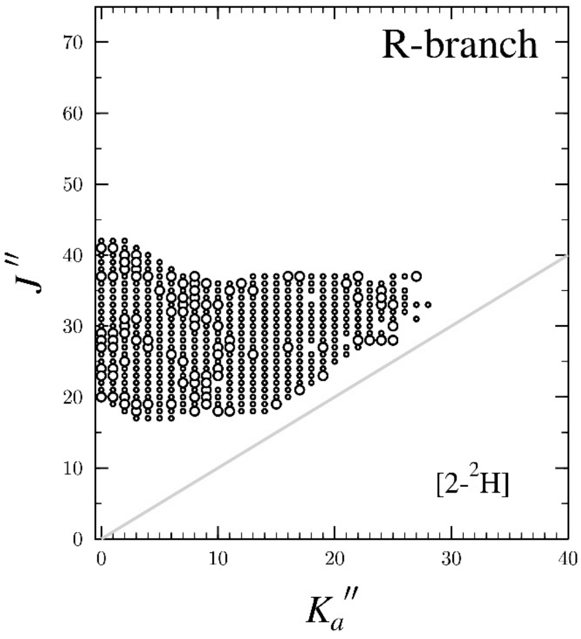

Figure S4 - Data distribution plot for the least-squares fit of spectroscopic data for the vibrational ground state of [2-^{2}H]-cyclopropanone. The size of the symbol is proportional to the value of $|(f_{obs.} - f_{calc.})/\delta f|$, where δf is the frequency measurement uncertainty of the relevant transition, and all quotient values are smaller than 3. Black circles represent millimeter-wave data.

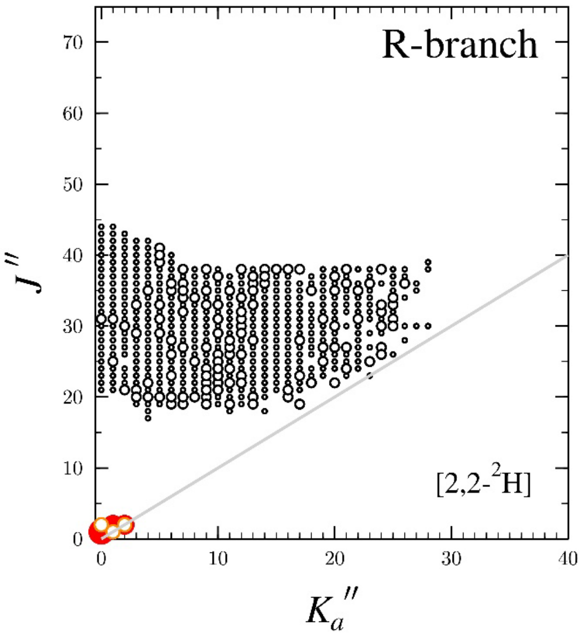

Figure S5 - Data distribution plot for the least-squares fit of spectroscopic data for the vibrational ground state of [2,2-^{2}H]-cyclopropanone. The size of the symbol is proportional to the value of $|(f_{obs.} - f_{calc.})/\delta f|$, where δf is the frequency measurement uncertainty of the relevant transition, and all quotient values are smaller than 3. Red circles indicate a quotient value greater than 3 for that measurement, black circles represent millimeter-wave data, and orange symbols represent microwave data from Pochan *et al*.[19]

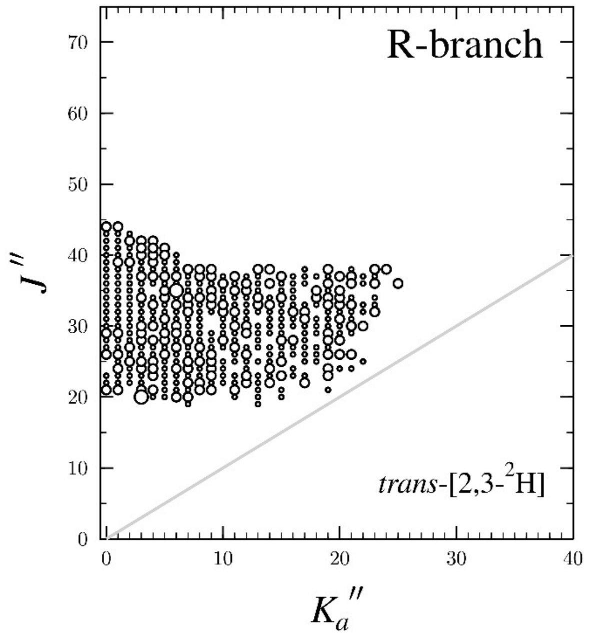

Figure S6 - Data distribution plot for the least-squares fit of spectroscopic data for the vibrational ground state of *trans*-[2,3-^{2}H]-cyclopropanone. The size of the symbol is proportional to the value of $|(f_{obs.} - f_{calc.})/\delta f|$, where δf is the frequency measurement uncertainty of the relevant transition, and all quotient values are smaller than 3. Black circles represent millimeter-wave data.

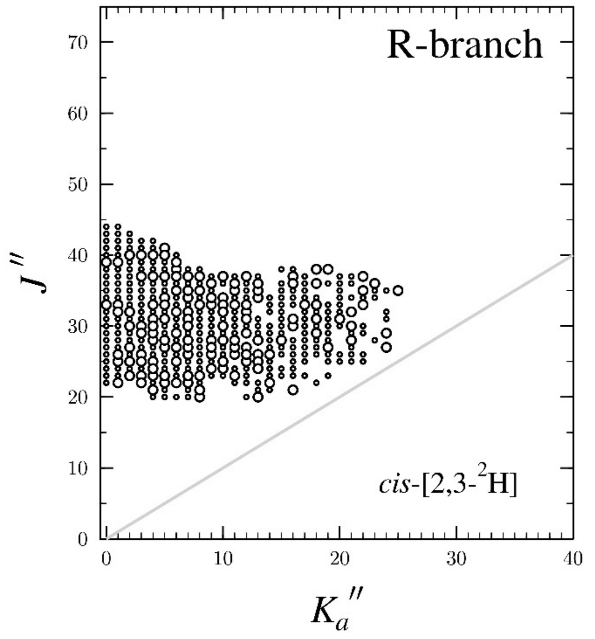

Figure S7 - Data distribution plot for the least-squares fit of spectroscopic data for the vibrational ground state of *cis*-[2,3-^{2}H]-cyclopropanone. The size of the symbol is proportional to the value of $|(f_{obs.} - f_{calc.})/\delta f|$, where δf is the frequency measurement uncertainty of the relevant transition, and all quotient values are smaller than 3. Black circles represent millimeter-wave data.

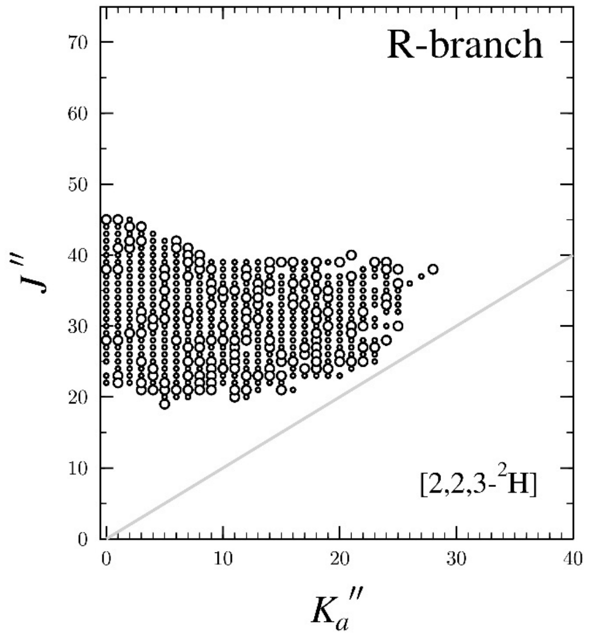

Figure S8 - Data distribution plot for the least-squares fit of spectroscopic data for the vibrational ground state of [2,2,3-^{2}H]-cyclopropanone. The size of the symbol is proportional to the value of $|(f_{obs.} - f_{calc.})/\delta f|$, where δf is the frequency measurement uncertainty of the relevant transition, and all quotient values are smaller than 3. Black circles represent millimeter-wave data.

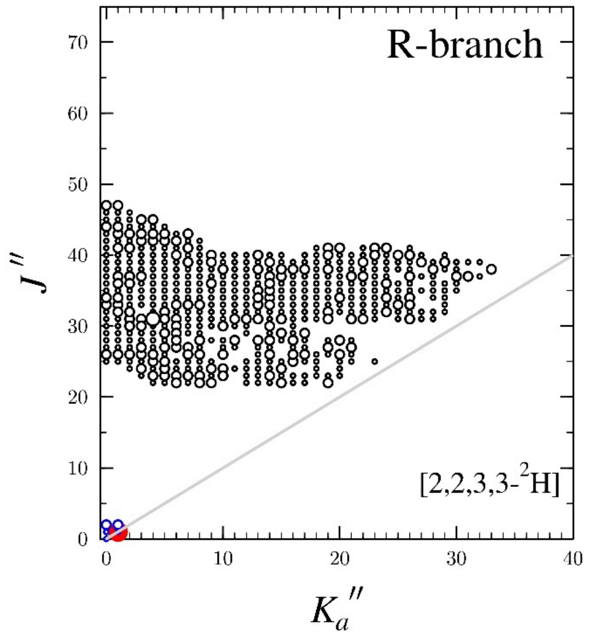

Figure S9 - Data distribution plot for the least-squares fit of spectroscopic data for the vibrational ground state of [2,2,3,3-^{2}H]-cyclopropanone. The size of the symbol is proportional to the value of $|(f_{obs.} - f_{calc.})/\delta f|$, where δf is the frequency measurement uncertainty of the relevant transition. Red circles indicate a quotient value greater than 3 for that measurement, black circles represent millimeter-wave data, while blue symbols represent microwave transitions recorded by the FT-microwave spectrometer.

Synthetic Procedures.

The synthesis of cyclopropanone from ketene and diazomethane has been reported previously (Scheme S1).[20-21] Laboratory scale diazomethane generators are commercially available.[22] The Wisconsin group previously reported the synthesis of ketene and deuteriated isotopologues.[11] Our initial syntheses of cyclopropanone were attempted using a ~5-fold excess of ketene. This ratio, however, led to cyclobutanone as the major product.[23-26] The ketene to diazomethane ratio was increased to a ~44-fold excess, which afforded cyclopropanone as the major product. As the solvent and excess ketene were removed, a white crystalline solid was formed, leading to the isolation of cyclopropanone polymer. The propensity of cyclopropanone to polymerize has been noted previously.[25, 27-28] The product had a high melting point that was variable between syntheses and the mass spectrum was consistent with a polymer of cyclopropanone with unknown end caps. Under vacuum, the solid polymer proved to reversibly depolymerize to a sufficient extent to produce a convenient equilibrium vapor pressure of monomer for rotational spectroscopy, even at ambient temperature. The slow release of monomer was sufficient to collect spectra for over 14 days in the Wisconsin spectrometer with an inlet manifold backing pressure of ~35 mTorr. In fact, the white polymeric material proved to be a very convenient and stable reservoir for cyclopropanone without any requirement of refrigeration, even allowing convenient shipping to other facilities.

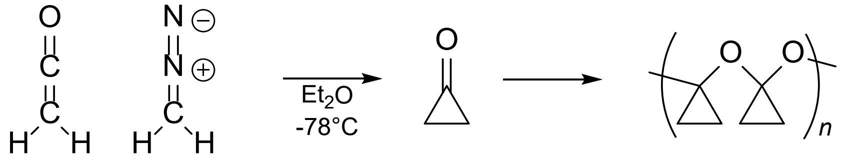

Scheme S1. Synthesis of cyclopropanone

Ketene. Synthesized by the pyrolysis of acetone (HPLC Grade) using a modified version of the Williams and Hurd ketene lamp,[29] described previously by the Wisconsin group (Scheme S2a).[11, 30] As ketene vapor is generated in the lamp, it flows through a water-cooled condenser, past a −78 °C cold finger condenser, and through a −78 °C cold trap to remove unreacted acetone and low volatility impurities (*e.g.* diketene). The purified ketene is then collected in a −130 °C cold trap. High volatility impurities were removed by holding the final cold trap under vacuum until a capacitance manometer reads ~3 mTorr. Ketene-d_x was synthesized using a mixed deuterio-/protio-solution of acetone-d_x, produced by a procedure modified from Paulsen and Cooke,[31] using acetone, D_2O, and lithium deuteroxide (LiOD). Without careful quantitation, a mixture of 150 g (1 eq) of HPLC grade acetone, 160 g (3 eq) of D_2O, and 0.1 mL saturated LiOD / D_2O solution is stirred for an hour then distilled, yielding approximately 50% deuterium-enriched acetone. Pyrolysis and purification of this mixture of acetone-d_x isotoplogues produced a mixture of ketene, [2-^{2}H]-ketene, and [2,2-^{2}H]-ketene (Scheme S2b). An independent sample of [2,2-^{2}H]-ketene (Scheme S2c) with high deuterium incorporation was generated by pyrolysis of acetone-d_6 (99.5%, Oakwood Chemical).

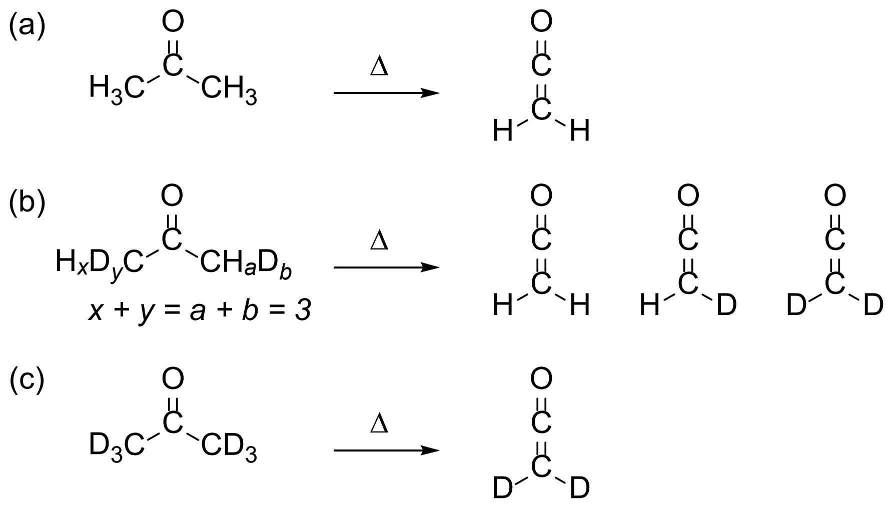

Scheme S2. Synthesis of ketene isotopologues

Diazomethane. Generated *in situ* via the reaction potassium hydroxide (2.5 g, 0.04 mol) dissolved in a mixture of water (4 mL, 0.22 mol) and 2-(2-ethoxyethoxy)ethanol (14 mL, 0.1 mol), and Diazald® (*N*-methyl-*N*-nitroso-*p*-toluenesulfonamide; Sigma-Aldrich) (2.53 g, 0.012 mol) in diethyl ether in an Aldrich Mini Diazald apparatus. The Diazald-ether solution is added dropwise to the aqueous hydroxide solution over the course of 1 h, generating diazomethane that is condensed on a cold finger as an ethereal solution and dripped into the cyclopropanone reaction vessel (see below). Diazomethane-d_x was synthesized by replacing H_2O with D_2O, yielding a mixture of CH_2N_2, $CHDN_2$, and CD_2N_2. Diazomethane with high deuterium incorporation was generated using 15 g of 40% NaOD in D_2O (purchased from Sigma Aldrich), 5 g of D_2O, 50 g 2-(2-ethoxyethoxy)ethanol-OD, and 2.1629 g of Diazald dissolved in anhydrous diethyl ether.

2-(2-Ethoxyethoxy)ethanol-OD was produced by enriching 2-(2-ethoxyethoxy)ethanol with D_2O in the presence of a catalytic amount of base. 100 g of 2-(2-Ethoxyethoxy)ethanol, 16 mL of D_2O, and 0.25 mL of $LiOD_{(sat.)}$ in D_2O were added to a 250-mL round-bottom flask and stirred at room temperature for approximately 30 min, then the D_2O was removed via distillation. A second, fresh 16-mL aliquot of D_2O was added to the round-bottom flask, and the mixture was stirred for approximately 30 min. D_2O was again removed via distillation, and this process was repeated until a total of six 16-mL aliquots of D_2O have been added, stirred, and distilled from the 2-(2-ethoxyethoxy)ethanol. At this point, 2-(2-ethoxyethoxy)ethanol was enriched to approximately 95% deuterium incorporation (−OD) and was purified via distillation to separate from LiOD.

Cyclopropanone / Oligo-cyclopropanone. A stir bar is added to a scratch-free heavy-wall 100-mL round-bottom flask with a single 19/22 Wheaton clear-seal neck, and the flask is connected to a vacuum manifold. The flask is placed under reduced atmosphere and cooled with

liquid nitrogen. Ketene (approximately 30 mL, 0.5 mol, or ~40 equivalents) is transferred to the flask via bulb-to-bulb vacuum distillation. While still in the liquid nitrogen Dewar, the flask containing ketene is backfilled with dry nitrogen to atmospheric pressure, removed from the manifold, and an Aldrich Mini Diazald apparatus is attached.[22] The hose barb of the Mini Diazald apparatus is connected to a nitrogen bubbler, and the condenser on the apparatus is cooled with dry ice and acetone. Potassium hydroxide (2.5 g) dissolved in 4 mL of deionized water, 14 mL of 2-(2-ethoxyethoxy)ethanol, and a stir bar are added to Diazald reaction vessel on the apparatus, and a pressure-equilibrating dropping funnel containing 2.53 g (0.012 mol) of Diazald dissolved in 22 mL of diethyl ether is inserted into clear-seal taper joint at the top of the reaction vessel. The top of the dropping funnel is sealed with a rubber septum.

The liquid nitrogen Dewar is removed and replaced with a dry ice-acetone bath Dewar. The stir plate beneath the round-bottom flask is set to a vigorous speed, and a small amount of ketene is observed to condense on the Diazald apparatus condenser. The Diazald reaction vessel is stirred and warmed to approximately 65 °C using a water bath. Over the course of approximately an hour the Diazald-ether solution is added to the Diazald reaction vessel, during which a yellow liquid is seen to condense before dripping into the round-bottom flask containing ketene. The dropping funnel is recharged with 10 mL of diethyl ether, and the ether is added to the Diazald reaction vessel until the condensing liquid is clear. The water bath is removed from the Diazald reaction vessel, and the round-bottom flask is stirred for 1 h while still cooled by the dry ice-acetone bath.

The dry ice-acetone Dewar is replaced with a liquid nitrogen Dewar, and the clear colorless liquid is observed to freeze. The Diazald apparatus is removed and the round-bottom flask is quickly attached to the vacuum manifold, then the flask is put under vacuum while still at liquid

nitrogen temperatures. The liquid nitrogen Dewar is removed, and while under static vacuum the reaction mixture is allowed to begin to thaw. Once the mixture is mostly liquid, the contents are subjected to vigorous stirring, and the valve connecting the flask to the vacuum manifold is slowly opened until effervescence is observed. The vacuum valve is left in this slightly opened position, and while this first fraction of ketene boils the flask is allowed to frost over through evaporative cooling, further attenuating the evaporation to reduce the risk of bumping.

After approximately 30 min, much of the liquid has been removed leaving a white slurry (Image S1), and the stirring is adjusted to a slower speed (Image S2). After approximately an additional 20 min, no liquid remains visible in the flask, which is now at room temperature. Stirring is stopped, and the valve to the vacuum manifold is closed. Still sealed, the sample is transferred to a vacuum manifold connected to a rotational spectrometer. The rotational spectrum of cyclopropanone is collected for several days, during which the sample is held at room temperature. After several days at room temperature the contents of the flask appear yellow and crystalline (Image S3), though in other experiments the product sometimes remains white. Because the samples were immediately used for rotational spectroscopy, the yield was not determined until after some of the material was consumed. Approximately 200 mg of the polycyclopropanone remained after all rotational spectroscopy had been completed.

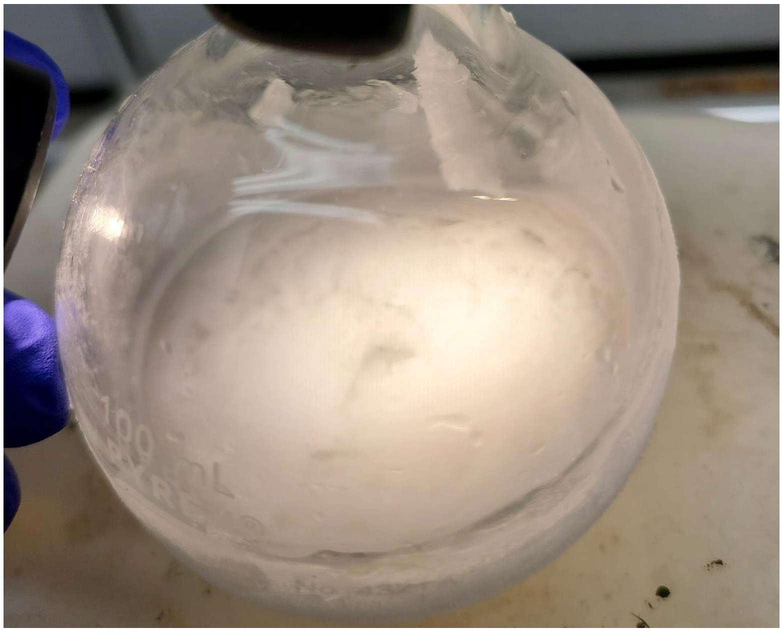

Image S1. White slurry after approximately 30 minutes of evaporation

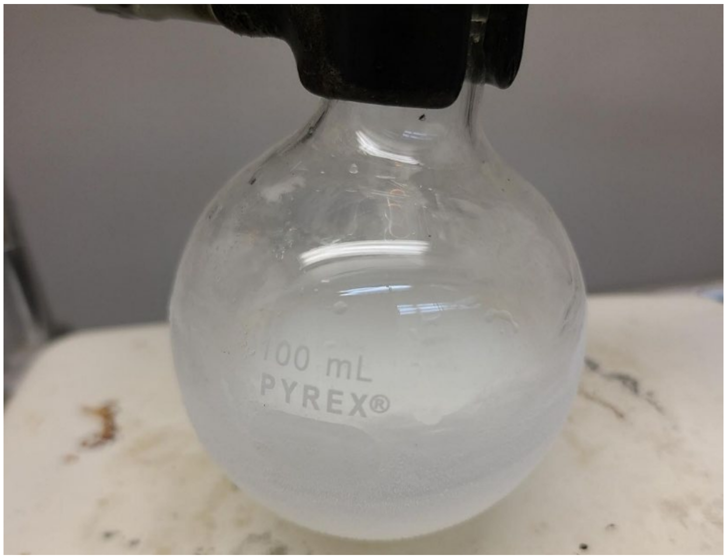

Image S2. White suspension in colorless liquid

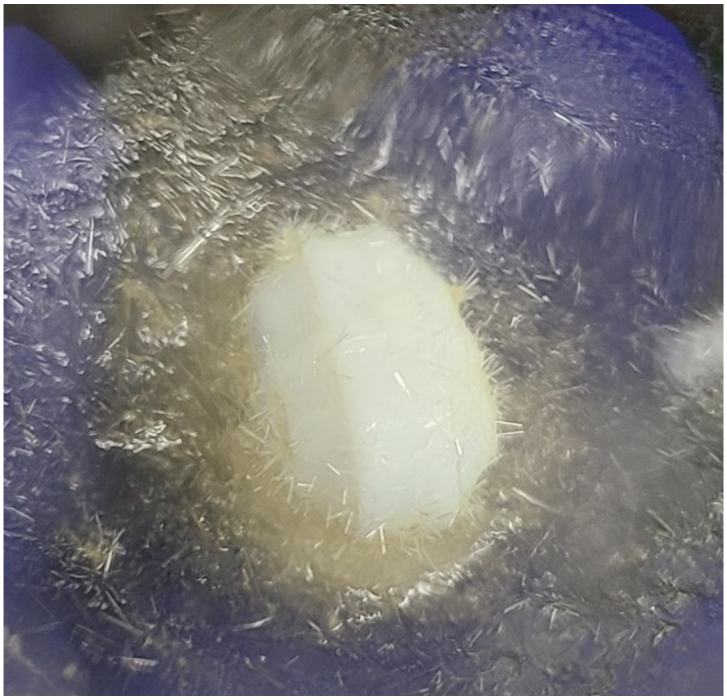

Image S3. Yellow crystals after several days at room temperature

The solid product is insoluble in water, chloroform, dichloromethane, trifluoroacetic acid, benzene, dimethyl sulfoxide, methanol, dimethylformamide, tetrahydrofuran, acetone, and hexafluoro-2-propanol, and darkens upon melting at 218 to 221 °C. A previous report of polycyclopropanone by Schaafsma *et al.* described a white solid with a melting point at 166.5-169.5 °C which was soluble in benzene and chloroform.[27] Quirk and Dunaway described lower molecular weight samples of polycyclopropanone (< 7,000 g/mol) as being soluble in methylene chloride, chloroform, toluene, and THF, while higher molecular weight samples were much less soluble.[28] A DSC trace of a 13,000 g/mol sample exhibited a sharp crystalline melting point of 167 °C. The white solid we created is stable in air at room temperature and off-gasses cyclopropanone under vacuum at room temperature, this was confirmed by collecting the

rotational spectrum of cyclopropanone that off-gassed from the white solid. Our samples have been used as convenient sources of cyclopropanone sometimes months after preparation and have been shipped to collaborators without apparent degradation.

Matrix-assisted laser desorption/ionization (MALDI) mass spectrum was conducted using a Bruker microflex LRF. A saturated solution of polycyclopropanone was prepared by wetting the solid with THF and drawing off a small amount of the liquid from the THF solid slurry. A matrix consisting of one μL THF saturated with the polycyclopropanone sample and one μL of a saturated solution of dithranol in THF was used. The mass spectrum showed several series of fragmentation peaks spaced 56 *m/z* apart, which can be interpreted as oligomers with similar end groups which vary in size by one unit of the monomer cyclopropanone (*m/z* = 56). Signals with *m/z* ratios of 318.1, 374.2, and 430.3 are consistent with 5, 6, and 7 cyclopropanone units, respectively, plus CH_3Na^+, but other interpretations are possible including 318.1 *m/z* possibly being caused by a closed ring of five cyclopropanone units with a potassium cation. The signals in the mass spectrum may not be representative of the bulk solid due to differences in solubility of the oligomers or possibly fragmentation during ionization. The MALDI mass spectra are in Figures S10 and S11, and Table S7 shows a table of calculated *m/z* ratios.

We suspect that the differences in solubility and melting temperature between our sample of polycyclopropanone and previous reports are associated with differences in chain length and/or end groups.[27-28] Structural uncertainties notwithstanding, this oligomeric / polymeric solid is a reliable source of the monomer cyclopropanone in a manner reminiscent of paraformaldehyde and formaldehyde.

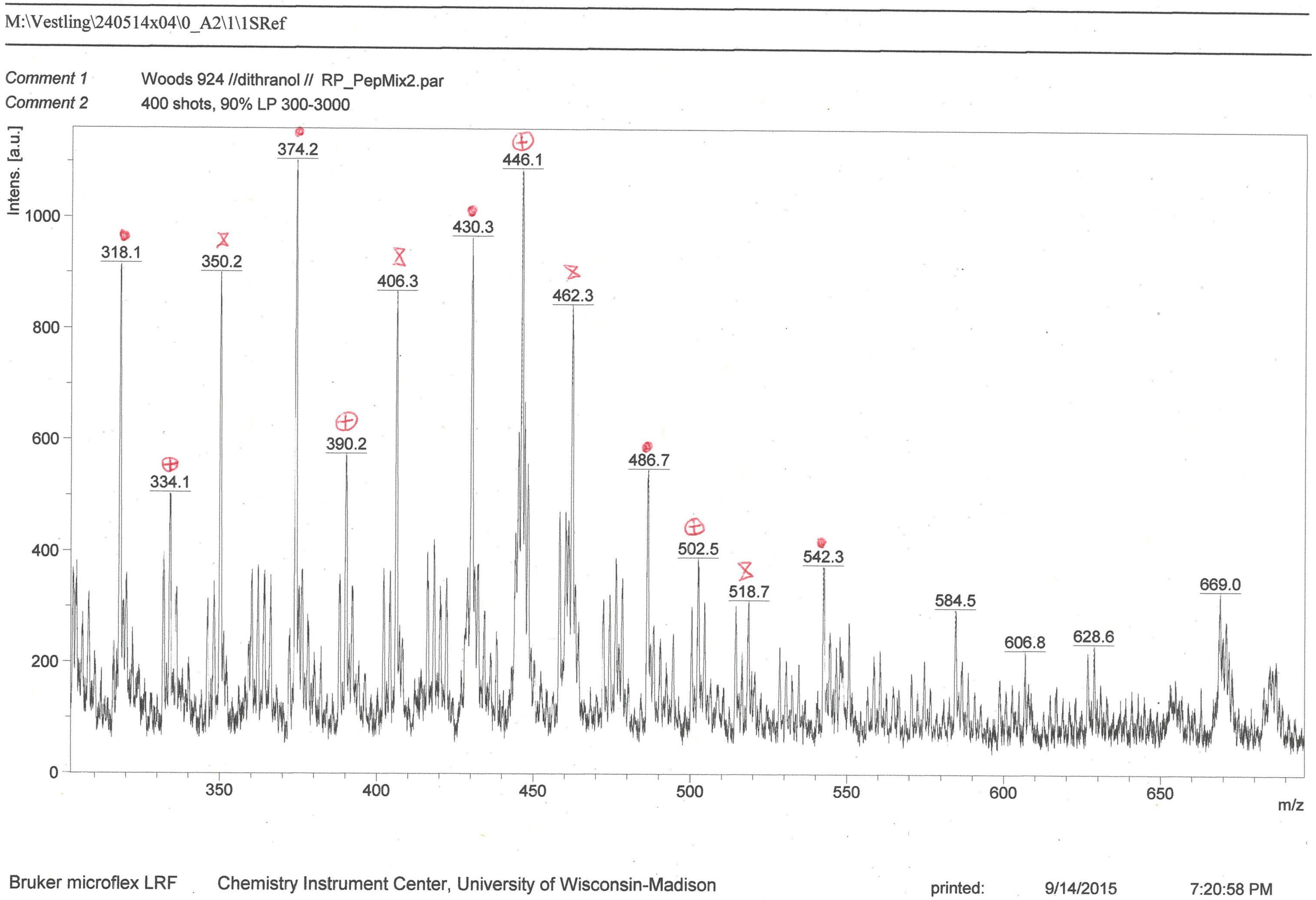

Figure S10. MALDI Mass spectrum of polycyclopropanone

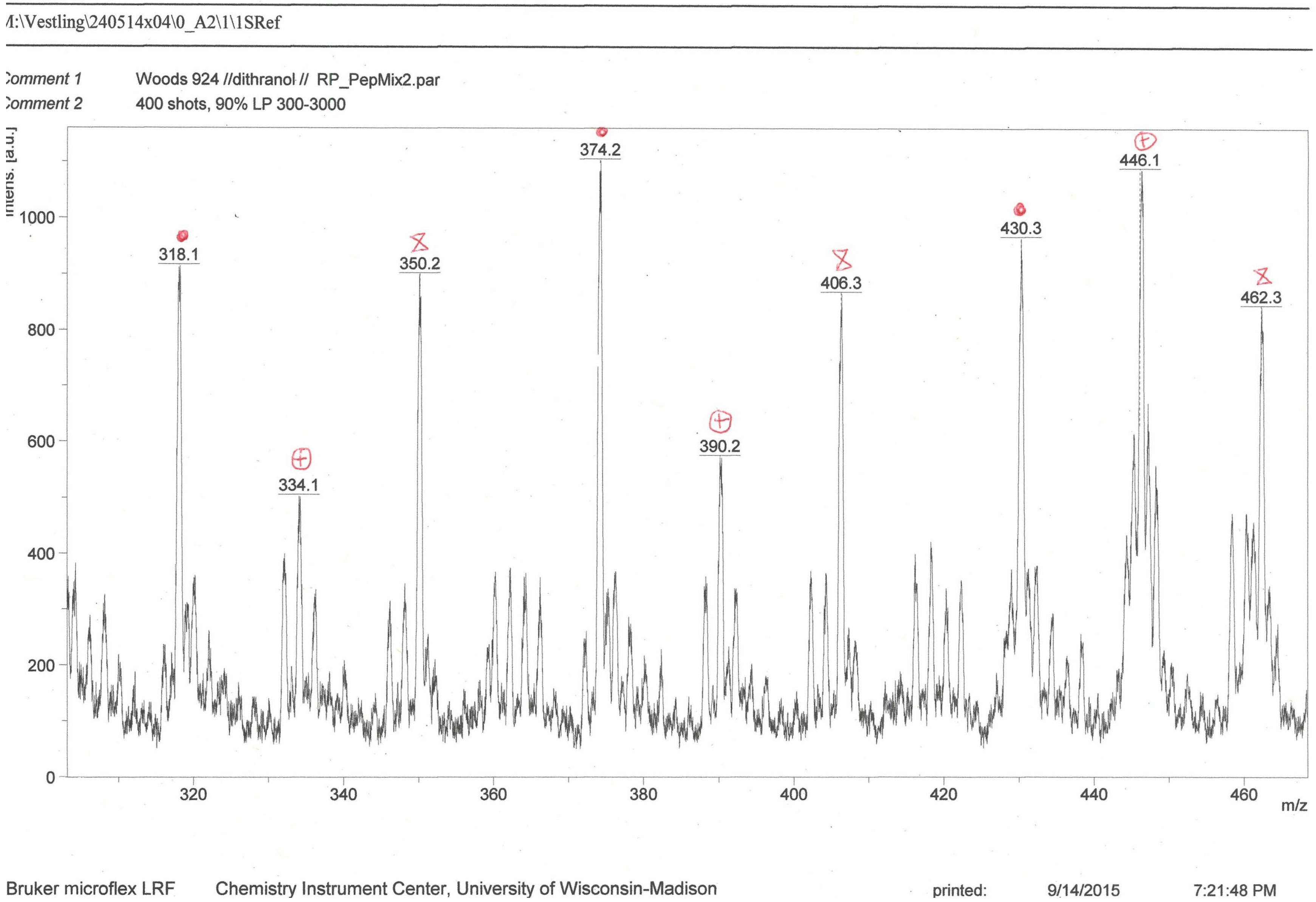

Figure S11. MALDI Mass spectrum of polycyclopropanone

Table S7. Various calculated *m/z* ratios

WOOD_924

**C_3H_4O**	56.0262						
**H_2O+Na+**	40.9999	CH_3O+Na+	54.0076	$CH_3O_2+Na^+$	70.0025	CH_3+Na^+	38.0127
**N**	H_2O + Na		CH3OH+Na		H-CH_2O_2+Na		CH_3+Na^+
**3**	209.0785		222.0862		238.0811		206.0913
**4**	265.1047		278.1124		294.1073		262.1175
**5**	321.1309		334.1386		350.1335		318.1437
**6**	377.1571		390.1648		406.1597		374.1699
**7**	433.1833		446.1910		462.1859		430.1961
**8**	489.2095		502.2172		518.2121		486.2223
**9**	545.2357		558.2434		574.2383		542.2485
**10**	601.2619		614.2696		630.2645		598.2747

END GROUPS

	CH_3O	$(C_3H_4O)_n$	H
	CH_2O_2		H
	CH_2		H

Cyclopropanone Deuterium/Hydrogen Isotopologues. Deuterium isotopologues were prepared from deuterium-enriched ketene and diazomethane precursors (Scheme S3). Synthesis of half deuterated ketene (Scheme S2b) and full deuterated ketene (Scheme S2c) from enriched acetone was achieved using a previously reported method.[11] Deuteration of diazomethane was achieved by using deuterated 2-(2-ethoxyethoxy)ethanol, sodium deuteroxide, and heavy water, as described in the Aldrich Diazald technical bulletin, and approximately 50% deuterium-enriched diazomethane was achieved by modifying this procedure.[22]

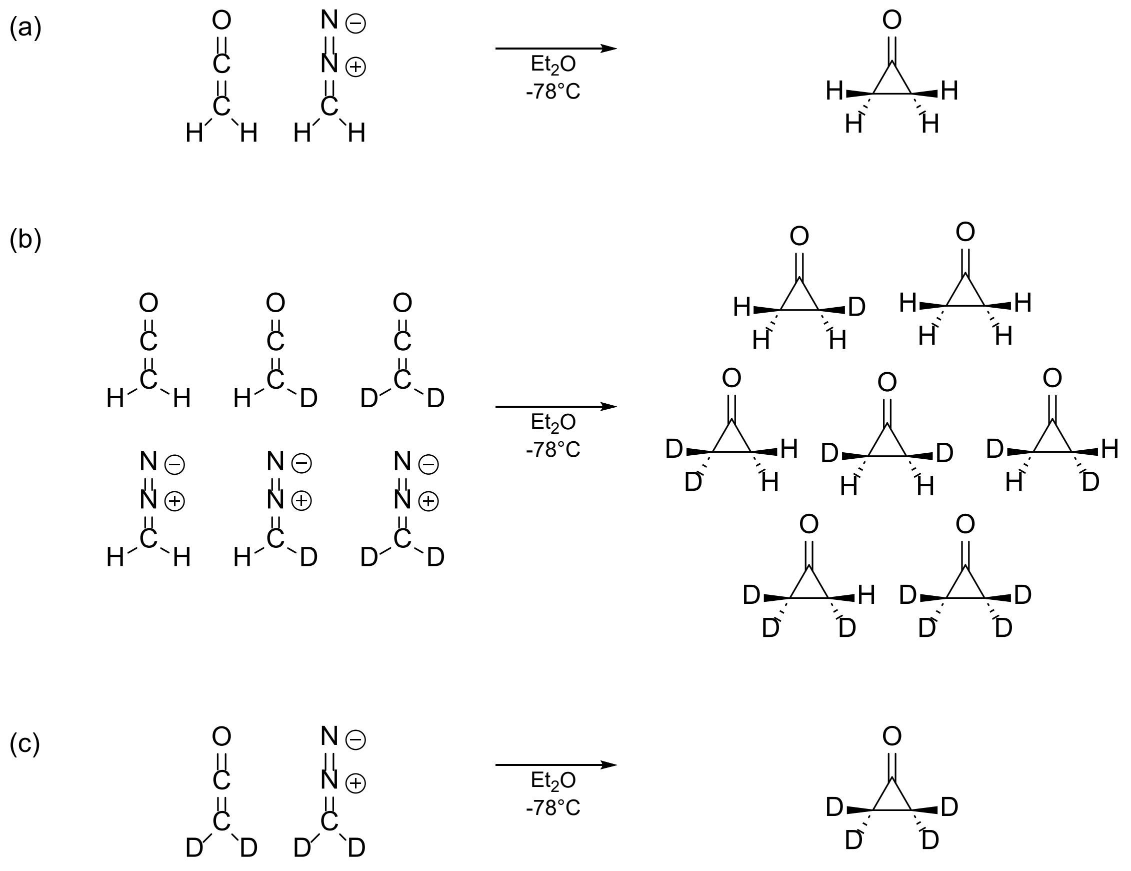

Scheme S3. Synthesis of cyclopropanone isotopologues

The following samples were prepared, enabling the detection of the specified isotopologues:

ketene-h_2 + diazomethane-h_2:	cyclopropanone + [1-^{13}C], [2-^{13}C], [^{18}O]
ketene-d_2 + diazomethane-d_2:	[2,2,3,3-^{2}H]-cyclopropanone
ketene-d_x + diazomethane-d_x:	cyclopropanone, [2-^{2}H], [2,2-^{2}H], [*cis*-2,3-^{2}H], [*trans*-2,3-^{2}H], [2,2,3-^{2}H], [2,2,3,3-^{2}H]

References

(1) Gordy, W.; Cook, R. L., *Microwave Molecular Spectra*. 3rd. ed.; Wiley Interscience: New York, 1984.

(2) Morgan, W. J.; Matthews, D. A.; Ringholm, M.; Agarwal, J.; Gong, J. Z.; Ruud, K.; Allen, W. D.; Stanton, J. F.; Schaefer, H. F., III, Geometric Energy Derivatives at the Complete Basis Set Limit: Application to the Equilibrium Structure and Molecular Force Field of Formaldehyde *J. Chem. Theory Comput.* **2018**, *14*, 1333−1350.

(3) Puzzarini, C.; Bloino, J.; Tasinato, N.; Barone, V., Accuracy and Interpretability: The Devil and the Holy Grail. New Routes across Old Boundaries in Computational Spectroscopy. *Chem. Rev.* **2019**, *119*, 8131-8191.

(4) Feller, D., The use of systematic sequences of wave functions for estimating the complete basis set, full configuration interaction limit in water *J. Chem. Phys.* **1993**, *98*, 7059-7071.

(5) Kutzelnigg, W.; Morgan, J. D., III, Rates of convergence of the partial-wave expansions of atomic correlation energies *J. Chem. Phys.* **1992**, *96*, 4484-4508.

(6) Halkier, A.; Helgaker, T.; Jørgensen, P.; Klopper, W.; Koch, H.; Olsen, J.; Wilson, A. K., Basis-set convergence in correlated calculations on Ne, N_2, and H_2O. *Chem. Phys. Lett.* **1998**, *286*, 243–252.

(7) Halkier, A.; Helgaker, T.; Jørgensen, P.; Klopper, W.; Olsen, J., Basis-set convergence of the energy in molecular Hartree–Fock calculations *Chem. Phys. Lett.* **1999**, *302*, 437-446.

(8) Helgaker, T.; Jørgensen, P.; Olsen, J., *Molecular Electronic-Structure Theory*. John Wiley & Sons: 2000.

(9) Bomble, Y. J.; Stanton, J. F.; Kállay, M.; Gauss, J., Coupled-cluster methods including noniterative corrections for quadruple excitations. *J. Chem. Phys.* **2005**, *123*, 054101.

(10) Heim, Z. N.; Amberger, B. K.; Esselman, B. J.; Stanton, J. F.; Woods, R. C.; McMahon, R. J., Molecular Structure Determination: Equilibrium Structure of Pyrimidine (m-$C_4H_4N_2$) from Rotational Spectroscopy (r_e^{SE}) and High-Level Ab Initio Calculation (r_e) Agree Within the Uncertainty of Experimental Measurement. *J. Chem. Phys.* **2020**, *152*, 104303.

(11) Smith, H. H.; Esselman, B. J.; Wood, S. A.; Stanton, J. F.; Woods, R. C.; McMahon, R. J., Improved Semi-Experimental Equilibrium Structure and High-Level Theoretical Structures of Ketene. *J. Chem. Phys.* **2023**, *158*, 244304.

(12) Cheng, L.; Gauss, J., Analytic energy gradients for the spin-free exact two-component theory using an exact block diagonalization for the one-electron Dirac Hamiltonian. *J. Chem. Phys.* **2011**, *135*, 084114.

(13) Liu, W.; Peng, D., Exact two-component Hamiltonians revisited. *J. Chem. Phys.* **2009**, *131*, 031104.

(14) Dyall, K. G., Interfacing relativistic and nonrelativistic methods. II. Investigation of a low-order approximation. *J. Chem. Phys.* **1998**, *109*, 4201-4208.

(15) Born, M.; Huang, K., *Dynamical Theory of Crystal Lattices*. Clarendon Press: Oxford, 1954.

(16) Handy, N. C.; Yamaguchi, Y.; Schaefer, H. F., III, The Diagonal Correction to the Born-Oppenheimer Approximation: Its Effect on the Singlet-Triplet Splitting of CH_2 and Other Molecular Effects. *J. Chem. Phys.* **1986**, *84*, 4481-4484.

(17) Gauss, J.; Puzzarini, C., Quantum-chemical calculation of Born-Oppenheimer breakdown parameters to rotational constants. *Mol. Phys.* **2010**, *108*, 269-277.

(18) Puzzarini, C.; Gauss, J., Quantum-chemical determination of Born-Oppenheimer breakdown parameters for rotational constants: the open-shell species CN, CO^+ and BO *Mol. Phys.* **2013**, *111*, 2204-2210.

(19) Pochan, J. M.; Baldwin, J. E.; Flygare, W. H., Microwave Spectrum and Structure of Cyclopropanone. *J. Am. Chem. Soc.* **1969**, *91*, 1896-1898.

(20) Turro, N. J., Cyclopropanones. *Acc. Chem. Res.* **1969**, *2*, 25-32.

(21) De Kimpe, N., Cyclopropanone. In *Encyclopedia of Reagents for Organic Synthesis (EROS)*, John Wiley & Sons, Ltd.: 2001.

(22) Diazald® and Diazomethane Generators, Sigma-Aldrich Co., 2007.

(23) Lipp, P.; Köster, R., Ein neuer Weg zum Cyclobutanon. *Ber. Dtsch. Chem. Ges. A/B* **1931**, *64*, 2823-2825.

(24) Semenow, D. A.; Cox, E. F.; Roberts, J. D., Small-Ring Compounds. XIV. Radioactive Cyclobutanone from Ketene and Diazomethane-$^{14}C^1$. *J. Am. Chem. Soc.* **1956**, *78*, 3221-3223.

(25) Turro, N. J.; Hammond, W. B., Cyclopropanone. *J. Am. Chem. Soc.* **1966**, *88*, 3672-3673.

(26) van Tilborg, W. J. M., The chemistry of small ring compounds. Part 22 absorptions of cyclopropanone in the infrared carbonyl region. *Tetrahedron Lett.* **1973**, *14*, 523-526.

(27) Schaafsma, S. E.; Steinberg, H.; de Boer, T. J., The Synthesis of Cyclopropanone. *Recl. Trav. Chim. Pays-Bas* **1966**, *85*, 1170-1172.

(28) Quirk, R. P.; Dunaway, J. H., Polycyclopropanone: Synthesis and Hydrogenolysis. In *Chemical Reactions on Polymers*, Benham, J. L.; Kinstle, J. F., Eds. American Chemical Society: Washington, DC, 1988; ACS Symposium Series Vol. 364, pp 141-152.

(29) Williams, J. W.; Hurd, C. D., An Improved Apparatus for the Laboratory Preparation of Keteme and Butadiene. *J. Org. Chem.* **1940**, *05*, 122-125.

(30) Wood, S. A.; Drier, T. O.; Esselman, B. J.; Woods, R. C.; McMahon, R. J., Ketene Lamp. *Fusion - Journal of the American Scientific Glassblowers Society* **2023**, *LXXI.4*, 21-29.

(31) Paulsen, P. J.; Cooke, W. D., Preparation of Deuterated Solvents for Nuclear Magnetic Resonance Spectrometry. *Anal. Chem.* **1963**, *35*, 1560.
